# Supplementary material for: Natural variability in bee brain size and symmetry revealed by micro-CT imaging and deep learning
Source: PLoS Comput Biol. 2023 Oct 2;19(10):e1011529. doi: 10.1371/journal.pcbi.1011529 (PMC10569549; doi:10.1371/journal.pcbi.1011529)
Supplement: S1 Fig — Segmentation results (left) and manually corrected results with segmentation errors highlighted (right) of Biomedisa’s deep neural network trained on 26 honey bee CT scans. (A) Correct segmentation without errors (bee ID 87, hive H4). (B) Partly flawed segmentation results with a typical outlier on the right edge of the image (bee ID 64, hive H5, segmentation accuracy: ME 97.6%, total 99.1%). (C) Significantly flawed segmentation result (bee ID 98, hive H6, total segmentation accuracy 87.7%). (DOCX) [file pcbi.1011529.s002.docx]

**S1 Fig. Segmentation results (left) and manually corrected results with segmentation errors highlighted (right) of Biomedisa’s deep neural network trained on 26 honey bee CT scans.** (**A**) Correct segmentation without errors (bee ID 87, hive H4). (**B**) Partly flawed segmentation results with a typical outlier on the right edge of the image (bee ID 64, hive H5, segmentation accuracy: ME 97.6%, total 99.1%). (**C**
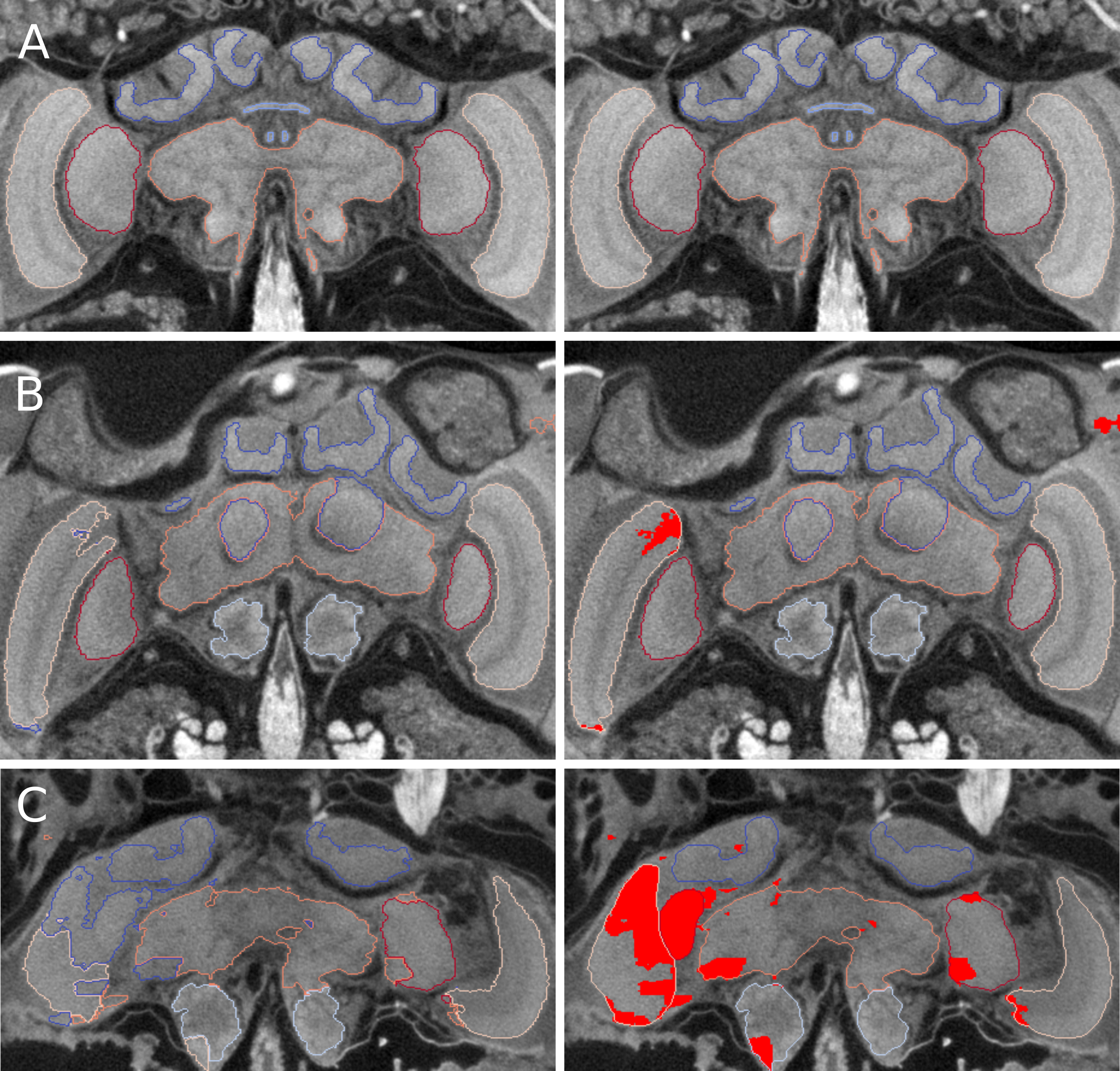
) Significantly flawed segmentation result (bee ID 98, hive H6, total segmentation accuracy 87.7%).
