# Supplementary material for: Natural variability in bee brain size and symmetry revealed by micro-CT imaging and deep learning
Source: PLoS Comput Biol. 2023 Oct 2;19(10):e1011529. doi: 10.1371/journal.pcbi.1011529 (PMC10569549; doi:10.1371/journal.pcbi.1011529)
Supplement: S2 Fig — Segmentation results (left) and manually corrected results with segmentation errors highlighted (right) of Biomedisa’s deep neural network trained on 13 bumblebee CT scans. (A) Correct segmentation without errors (ID 93). (B) Partly flawed segmentation results, with CX almost not recognised (ID 81, segmentation accuracy: CX 20.4%, total 97.9%). (C) Significantly flawed segmentation result (ID 57, total segmentation accuracy 88.3%). (DOCX) [file pcbi.1011529.s003.docx]

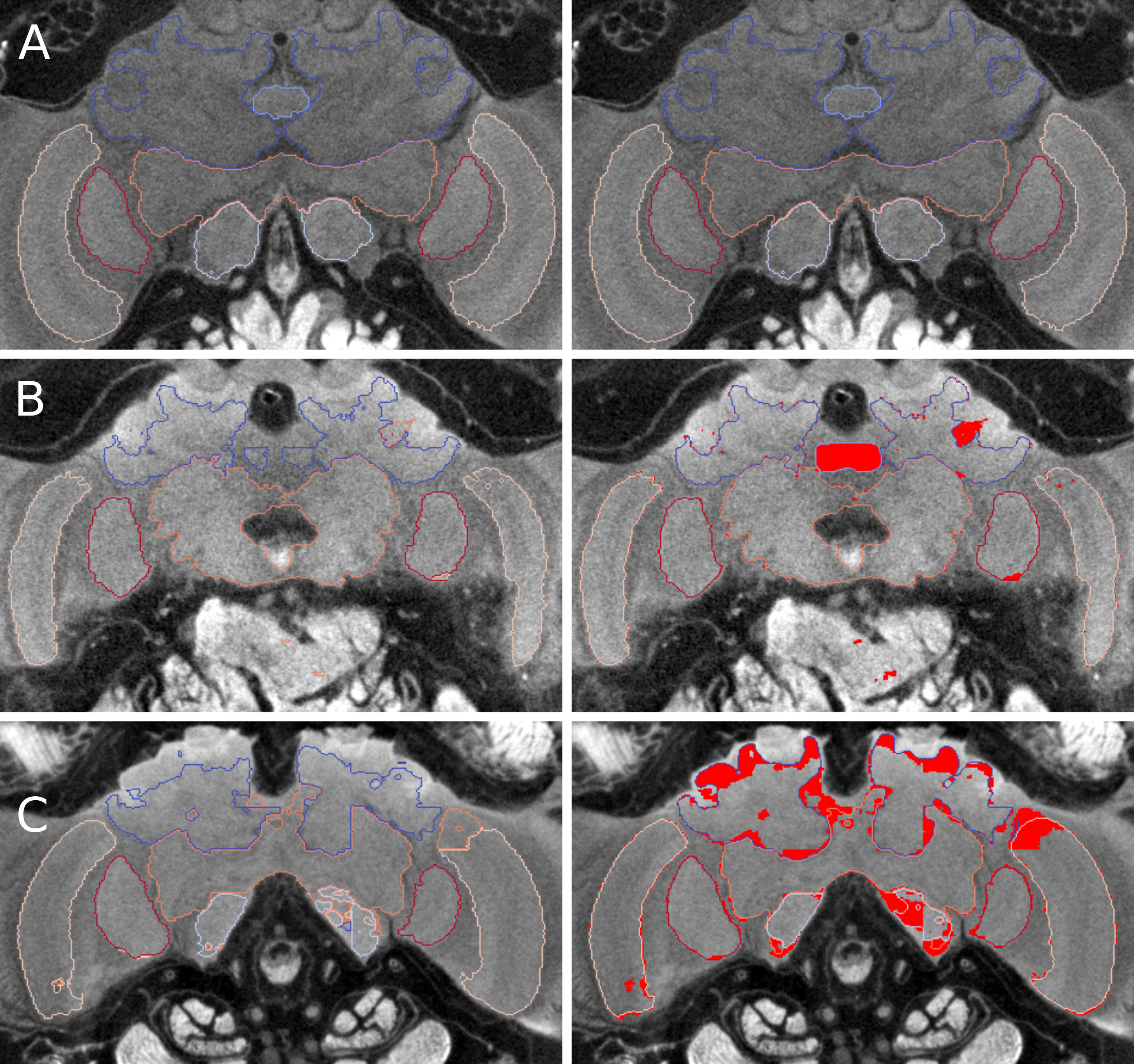
**S2 Fig. Segmentation results (left) and manually corrected results with segmentation errors highlighted (right) of Biomedisa’s deep neural network trained on 13 bumblebee CT scans.** (**A**) Correct segmentation without errors (ID 93). (**B**) Partly flawed segmentation results, with CX almost not recognised (ID 81, segmentation accuracy: CX 20.4%, total 97.9%). (**C**) Significantly flawed segmentation result (ID 57, total segmentation accuracy 88.3%).
