# Supplementary material for: Natural variability in bee brain size and symmetry revealed by micro-CT imaging and deep learning
Source: PLoS Comput Biol. 2023 Oct 2;19(10):e1011529. doi: 10.1371/journal.pcbi.1011529 (PMC10569549; doi:10.1371/journal.pcbi.1011529)
Supplement: S3 Fig — Results from 3 to 26 three-dimensional bumblebee training images, utilising 20 three-dimensional validation images during training. The final evaluation is performed on 24 three-dimensional test images. The networks pre-trained on honey bee image data and fine-tuned to bumblebee image data (green), showing improved performance for up to 7 three-dimensional training images compared to training from scratch with initially standard normal distributed weights (red). (DOCX) [file pcbi.1011529.s004.docx]

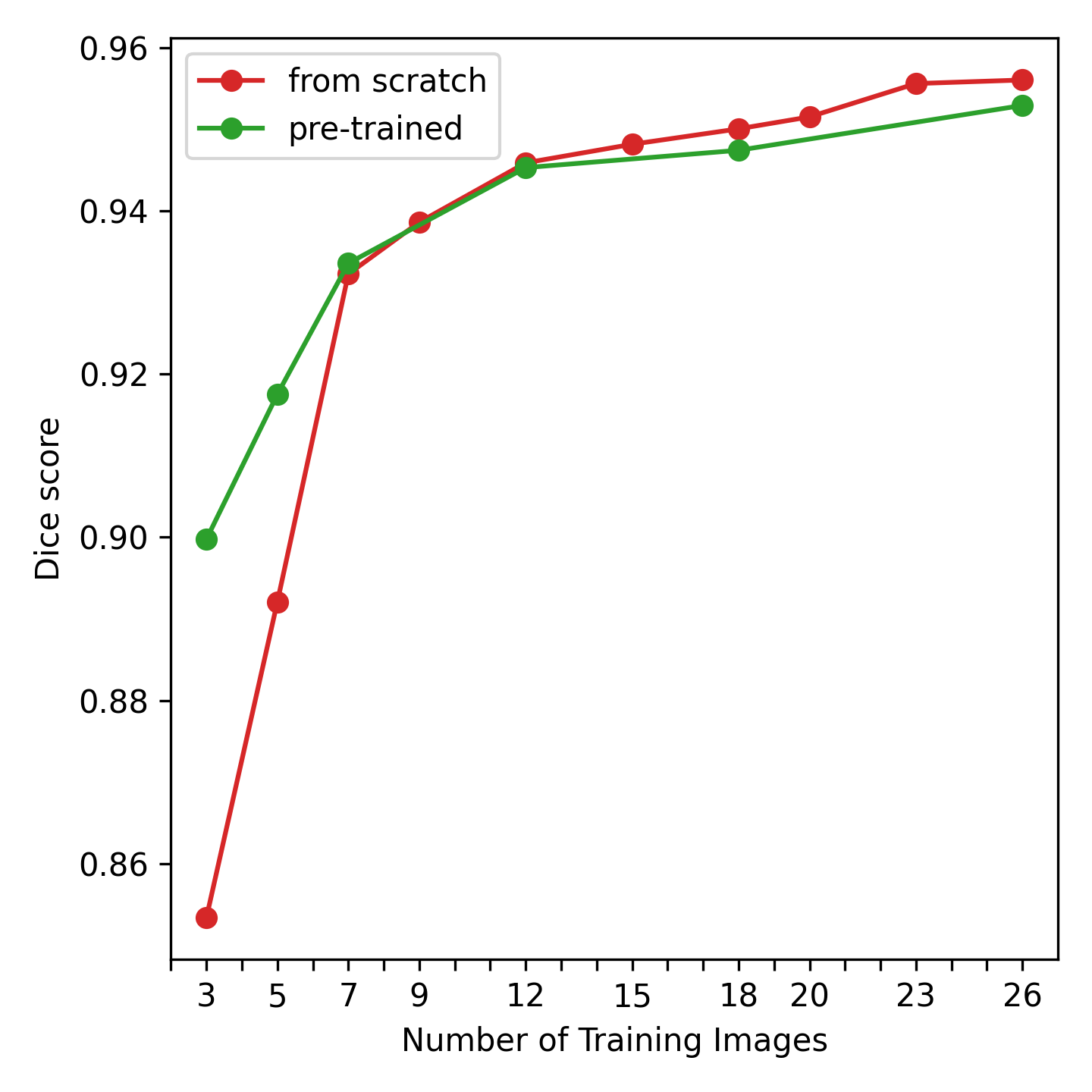
**S3 Fig. Fine-tuning neural network on bumblebee data for an increasing number of 3D training images.** Results from 3 to 26 three-dimensional bumblebee training images, utilising 20 three-dimensional validation images during training. The final evaluation is performed on 24 three-dimensional test images. The networks pre-trained on honey bee image data and fine-tuned to bumblebee image data (*green*), showing improved performance for up to 7 three-dimensional training images compared to training from scratch with initially standard normal distributed weights (*red*).
