# Supplementary material for: Natural variability in bee brain size and symmetry revealed by micro-CT imaging and deep learning
Source: PLoS Comput Biol. 2023 Oct 2;19(10):e1011529. doi: 10.1371/journal.pcbi.1011529 (PMC10569549; doi:10.1371/journal.pcbi.1011529)
Supplement: S4 Fig — Results from 13 three-dimensional training images and 20 three-dimensional validation images show the progress of accuracy in bumblebee data. While the standard accuracy of the training data (red) continues to improve over the course of training, the standard accuracy (green) and the Dice score (blue) of the validation data level off at their maximums of 0.975 after 154 epochs (orange) and 0.953 after 129 epochs (cyan), respectively. Dice score for training data is not available on Biomedisa. (DOCX) [file pcbi.1011529.s005.docx]

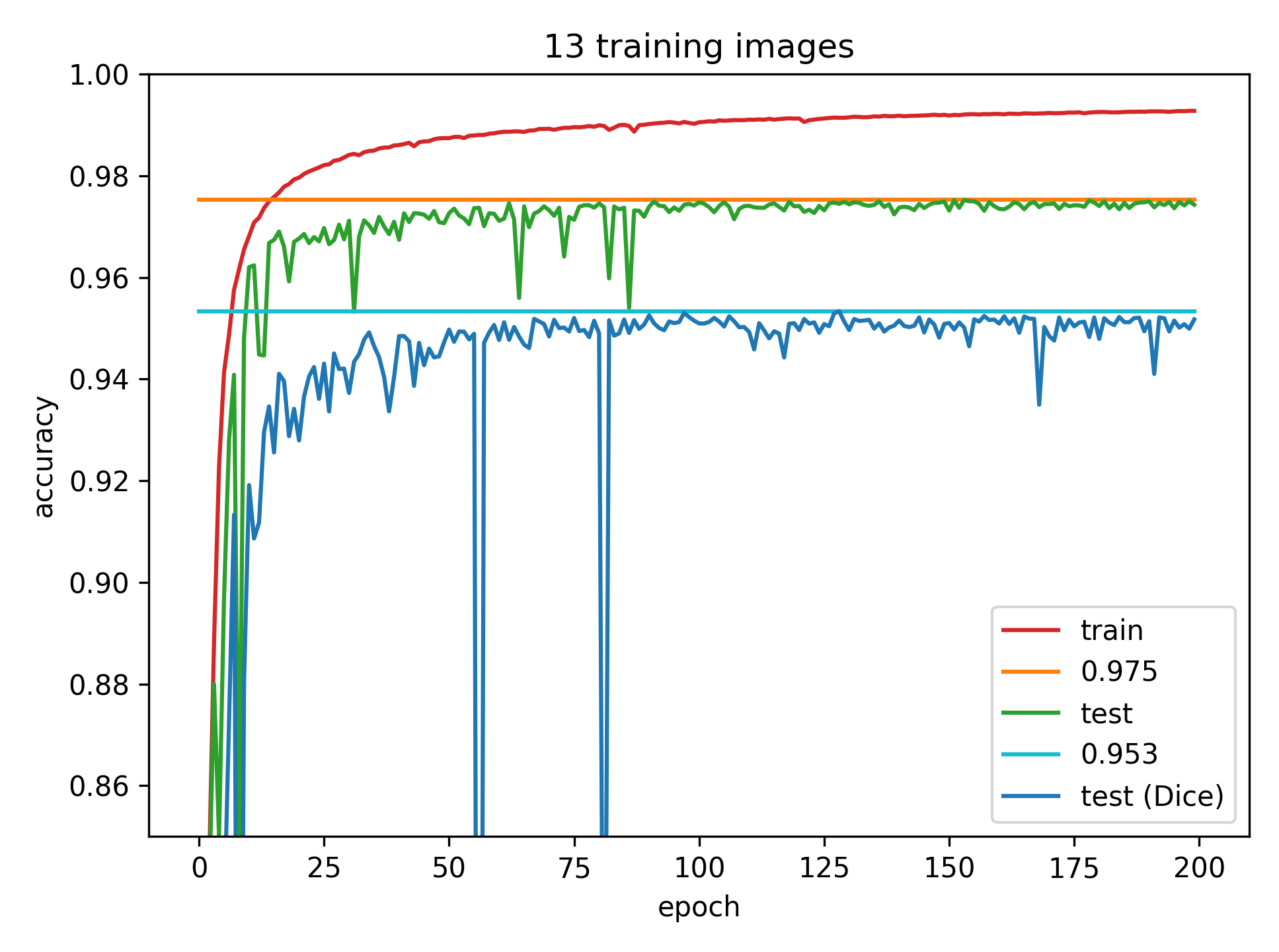
**S4 Fig. Bumblebee training and validation data accuracy.** Results from 13 three-dimensional training images and 20 three-dimensional validation images show the progress of accuracy in bumblebee data. While the standard accuracy of the training data (*red*) continues to improve over the course of training, the standard accuracy (*green*) and the Dice score (*blue*) of the validation data level off at their maximums of 0.975 after 154 epochs (*orange*) and 0.953 after 129 epochs (*cyan*), respectively. Dice score for training data is not available on Biomedisa.
