# Supplementary material for: Natural variability in bee brain size and symmetry revealed by micro-CT imaging and deep learning
Source: PLoS Comput Biol. 2023 Oct 2;19(10):e1011529. doi: 10.1371/journal.pcbi.1011529 (PMC10569549; doi:10.1371/journal.pcbi.1011529)
Supplement: S5 Fig — Results from 13 three-dimensional training images and 20 three-dimensional validation images. While the loss of the training data (red) continues to decrease over the course of training, the loss of the validation data (green) begins to increase marginally after reaching its minimum at 0.08 (orange). (DOCX) [file pcbi.1011529.s006.docx]

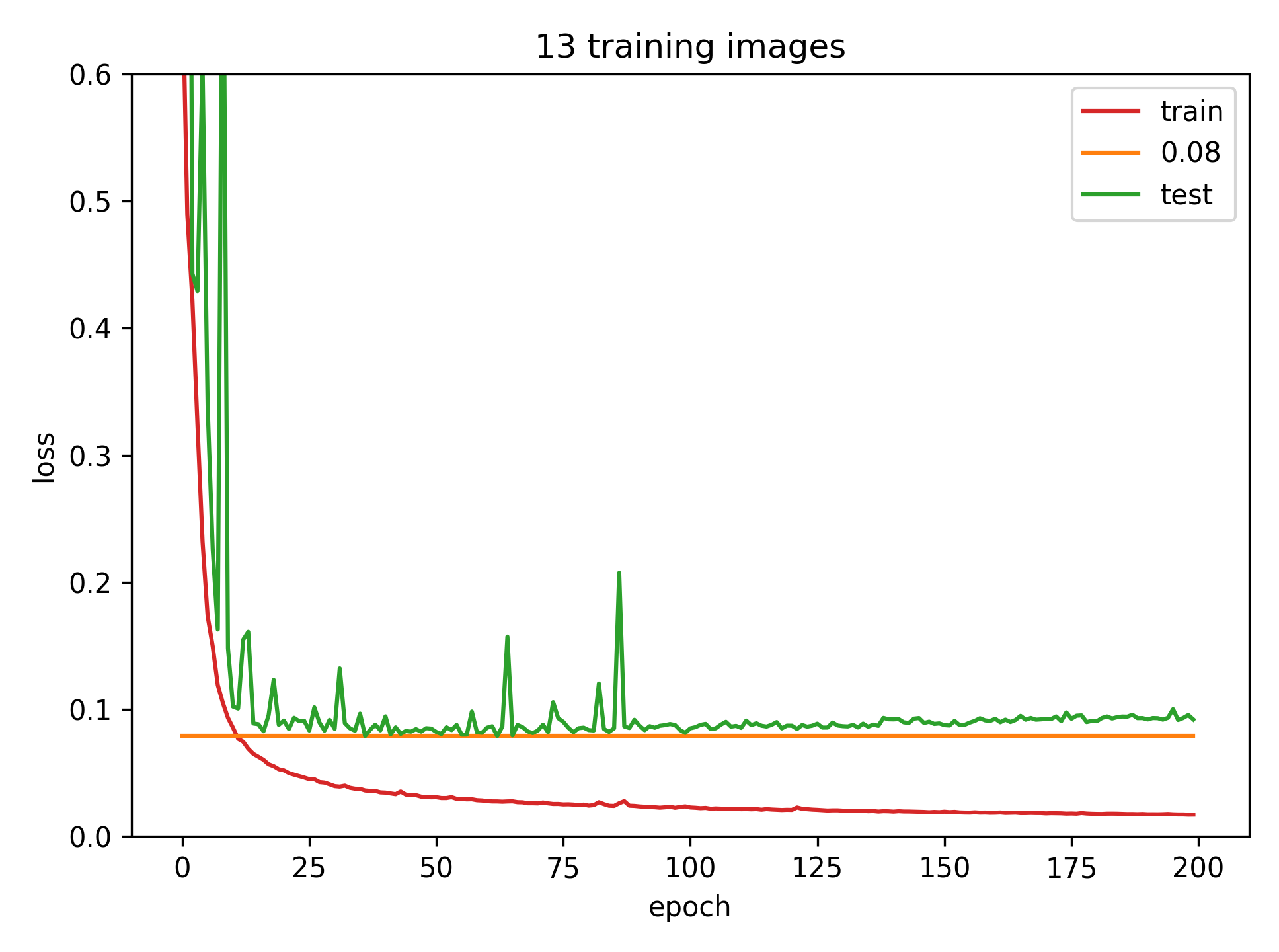
**S5 Fig. Bumblebee training and validation loss.** Results from 13 three-dimensional training images and 20 three-dimensional validation images. While the loss of the training data (*red*) continues to decrease over the course of training, the loss of the validation data (*green*) begins to increase marginally after reaching its minimum at 0.08 (*orange*).
