# Supplementary material for: Natural variability in bee brain size and symmetry revealed by micro-CT imaging and deep learning
Source: PLoS Comput Biol. 2023 Oct 2;19(10):e1011529. doi: 10.1371/journal.pcbi.1011529 (PMC10569549; doi:10.1371/journal.pcbi.1011529)
Supplement: S6 Fig — Results from 26 three-dimensional training images and 30 three-dimensional validation images show the progress of accuracy in honey bee data. While the standard accuracy of the training data (red) continues to improve over the course of training, the standard accuracy (green) and the Dice score (blue) of the validation data level off at their maximums of 0.985 after 197 epochs (orange) and 0.969 after 123 epochs (cyan), respectively. Please note that the Dice score for training data is not available on Biomedisa. (DOCX) [file pcbi.1011529.s007.docx]

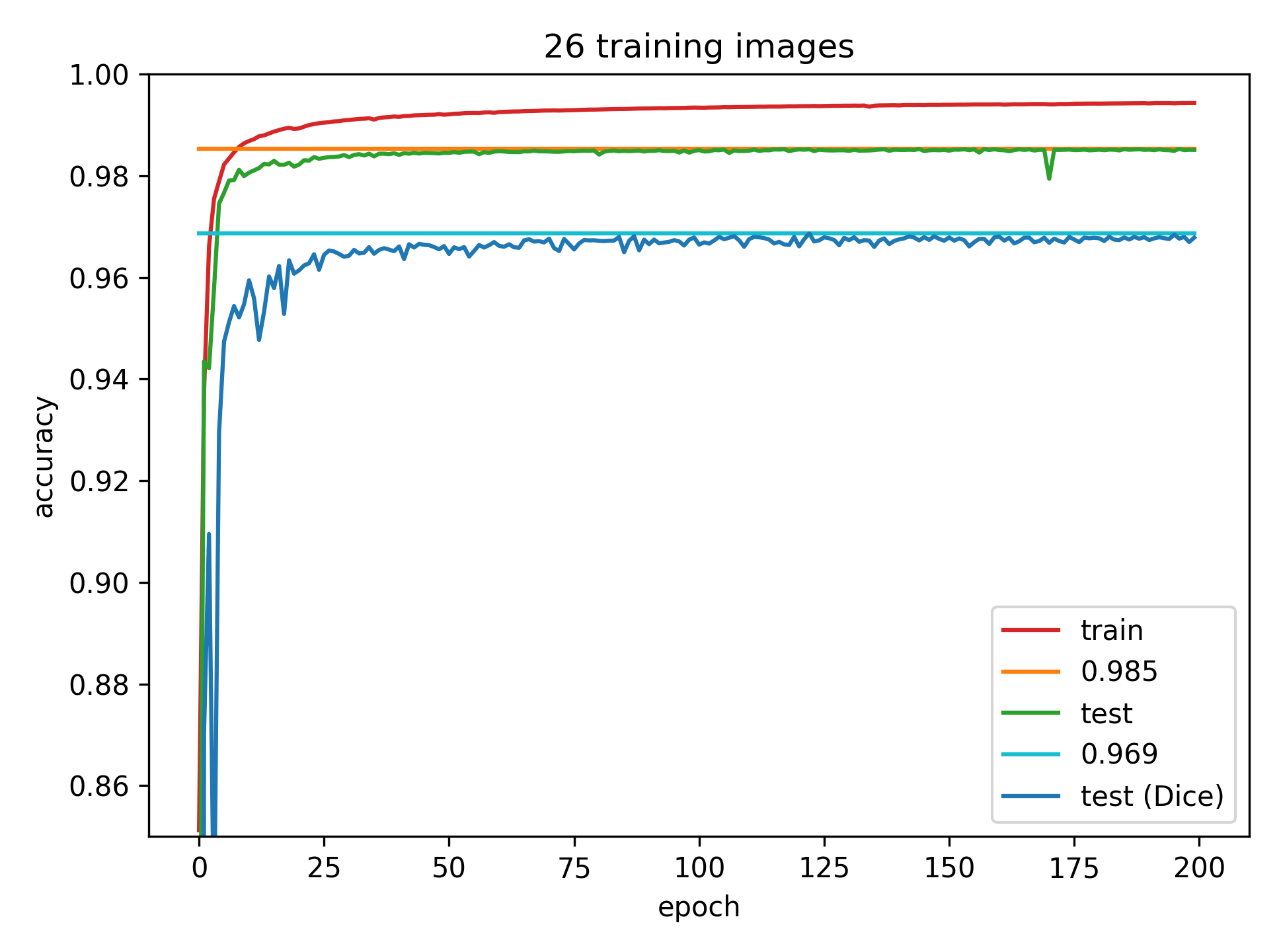
**S6 Fig. Honey bee training and validation data accuracy.** Results from 26 three-dimensional training images and 30 three-dimensional validation images show the progress of accuracy in honey bee data. While the standard accuracy of the training data (*red*) continues to improve over the course of training, the standard accuracy (*green*) and the Dice score (*blue*) of the validation data level off at their maximums of 0.985 after 197 epochs (*orange*) and 0.969 after 123 epochs (*cyan*), respectively. Please note that the Dice score for training data is not available on Biomedisa.
