# Supplementary material for: Natural variability in bee brain size and symmetry revealed by micro-CT imaging and deep learning
Source: PLoS Comput Biol. 2023 Oct 2;19(10):e1011529. doi: 10.1371/journal.pcbi.1011529 (PMC10569549; doi:10.1371/journal.pcbi.1011529)
Supplement: S8 Fig — (A) Antennal lobes (AL). (B) Mushroom bodies (MB). (C) Optic lobes (OL). (D) Medullae (ME). (E) Lobulae (LO). (F) Central complex (CX). (G) Other neuropils (OTH). Regression lines displayed with 95% confidence intervals. Pearson correlation coefficient (r) and p-value are given. Strong correlations (r>0.40) and significant correlations (p<0.05) are displayed in bold. (H) Linear correlations for the different neuropils relative volume (y-axis not given: differs for each neuropil). The grey dashed line indicates true isometric correlation (slope = 1). (DOCX) [file pcbi.1011529.s009.docx]

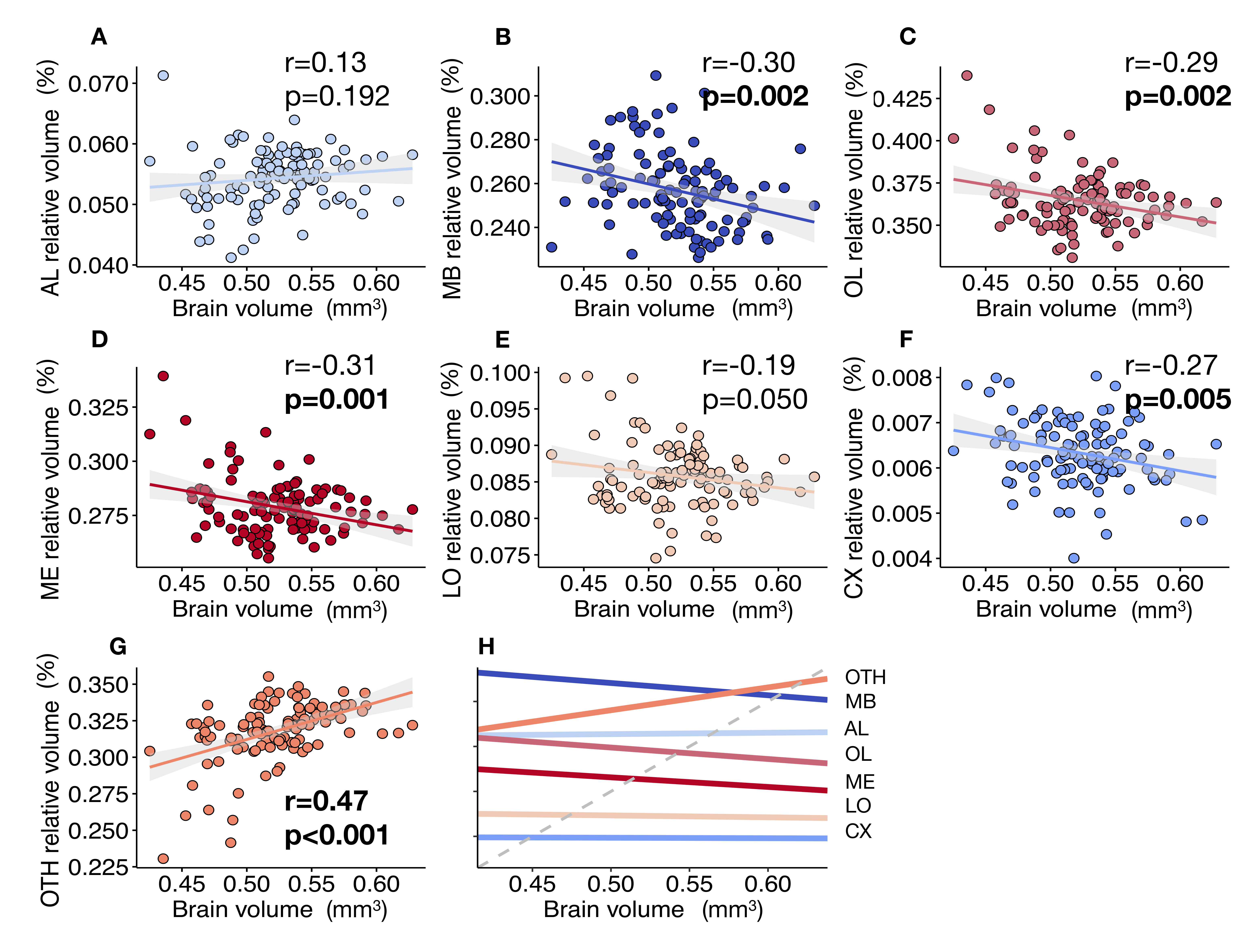
**S8 Fig. Correlation between relative volumes of neuropils and total brain volume (mm^3^) for honey bees (N=110).** (**A**) Antennal lobes (AL). (**B**) Mushroom bodies (MB). (**C**) Optic lobes (OL). (**D**) Medullae (ME). (**E**) Lobulae (LO). (**F**) Central complex (CX). (**G**) Other neuropils (OTH). Regression lines displayed with 95% confidence intervals. Pearson correlation coefficient (r) and p-value are given. Strong correlations (r>0.40) and significant correlations (p<0.05) are displayed in bold. (**H**) Linear correlations for the different neuropils relative volume (y-axis not given: differs for each neuropil). The grey dashed line indicates true isometric correlation (slope=1).
