# Supplementary material for: Natural variability in bee brain size and symmetry revealed by micro-CT imaging and deep learning
Source: PLoS Comput Biol. 2023 Oct 2;19(10):e1011529. doi: 10.1371/journal.pcbi.1011529 (PMC10569549; doi:10.1371/journal.pcbi.1011529)
Supplement: S11 Fig — AL and OL (N = 110 honey bees, N = 77 bumblebees), MB and AL and MB and OL (N = 59 honey bees, N = 36 bumblebees). (DOCX) [file pcbi.1011529.s012.docx]

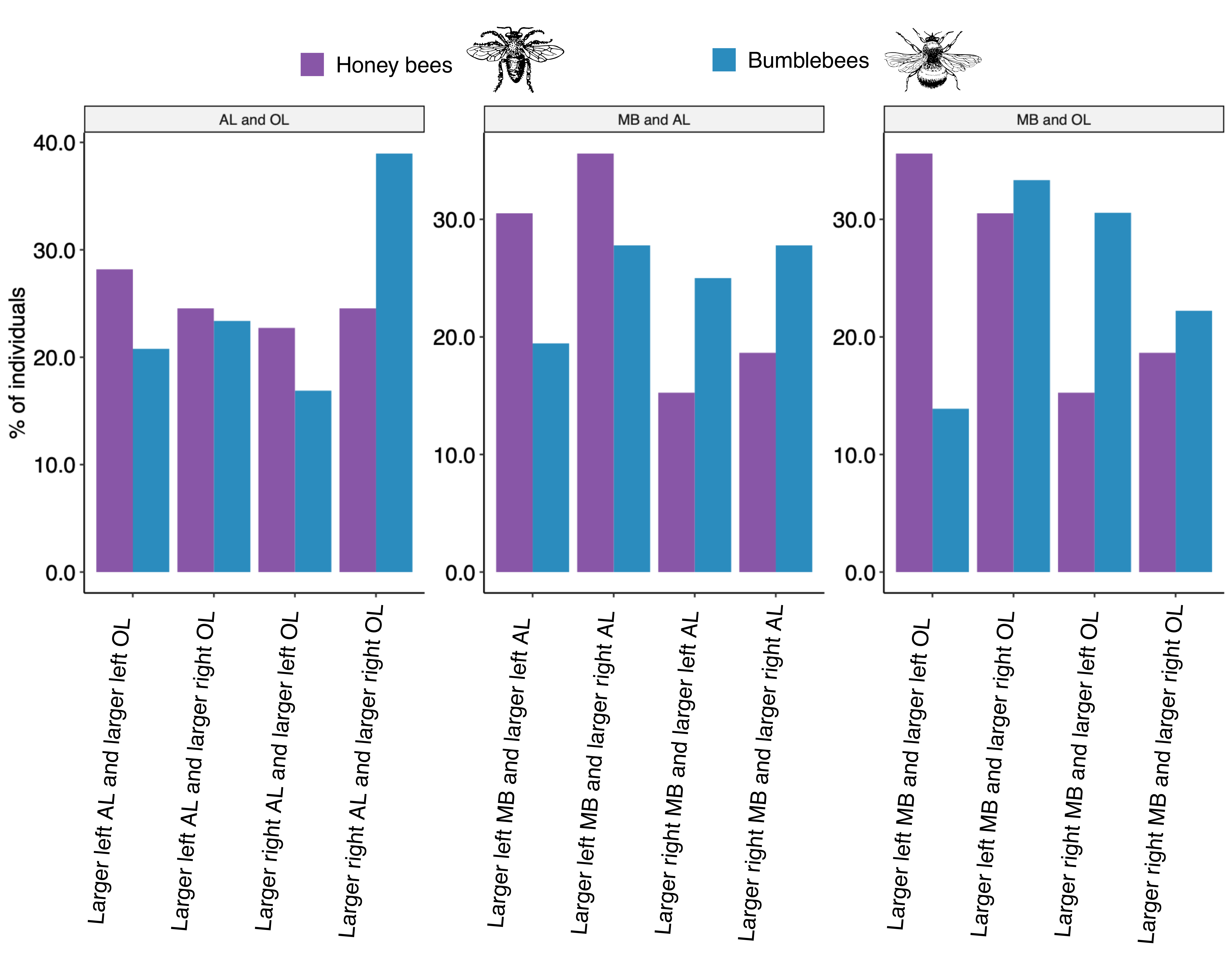
**S11 Fig. Percentage of individuals per asymmetry categories for honey bees (*purple*) and bumblebees (*blue*).** AL and OL (N=110 honey bees, N=77 bumblebees), MB and AL and MB and OL (N=59 honey bees, N=36 bumblebees).
