# Supplementary material for: Natural variability in bee brain size and symmetry revealed by micro-CT imaging and deep learning
Source: PLoS Comput Biol. 2023 Oct 2;19(10):e1011529. doi: 10.1371/journal.pcbi.1011529 (PMC10569549; doi:10.1371/journal.pcbi.1011529)
Supplement: S1 Table — Outliers were automatically removed (see “Methods”). Last column: amount of manual correction required (Error in % of Dice score). Brain areas are labelled using the same abbreviations as in Fig 2. For some of the performance tests of the automatic segmentation (yellow), the 84 three-dimensional honey bee test images were split into 30 three-dimensional validation images and 54 three-dimensional test images (see “Methods”). Additionally, for honey bees that were initially scanned upside down, the 3D image data was flipped along the z-axis (yellow). It is important to note that all image data utilised in the study is three-dimensional. (DOCX) [file pcbi.1011529.s013.docx]

| **S1 Table. Average Dice scores of semi-automatic and automatic segmentation results of bumblebee (row 2) and honey bee brains (rows 3–12).** Outliers were automatically removed (see “Methods”). Last column: amount of manual correction required (Error in % of Dice score). Brain areas are labelled using the same abbreviations as in Fig 2. For some of the performance tests of the automatic segmentation (*yellow*), the 84 three-dimensional honey bee test images were split into 30 three-dimensional validation images and 54 three-dimensional test images (see “Methods”). Additionally, for honey bees that were initially scanned upside down, the 3D image data was flipped along the z-axis (yellow). It is important to note that all image data utilised in the study is three-dimensional. | | | | | | | | |
| --- | --- | --- | --- | --- | --- | --- | --- | --- |
| **Dataset** | **AL** | **MB** | **ME** | **LO** | **CX** | **OTH** | **Total** | **Error** |
| 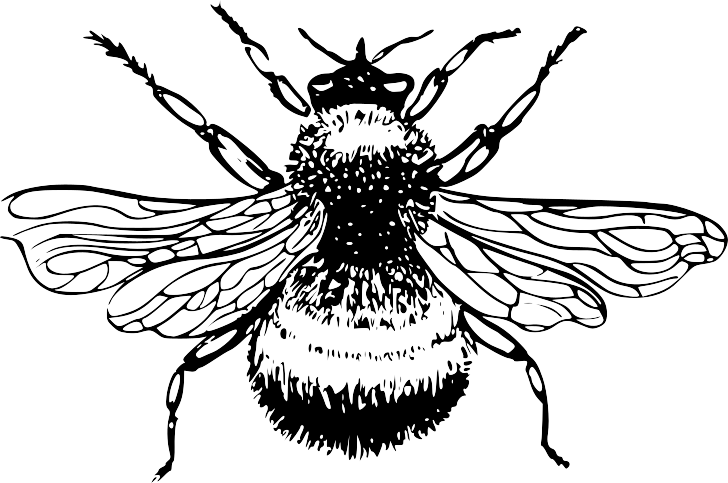**64 test images** | 0.983 | 0.985 | 0.983 | 0.982 | 0.838 | 0.980 | **0.983** | **1.7%** |
| 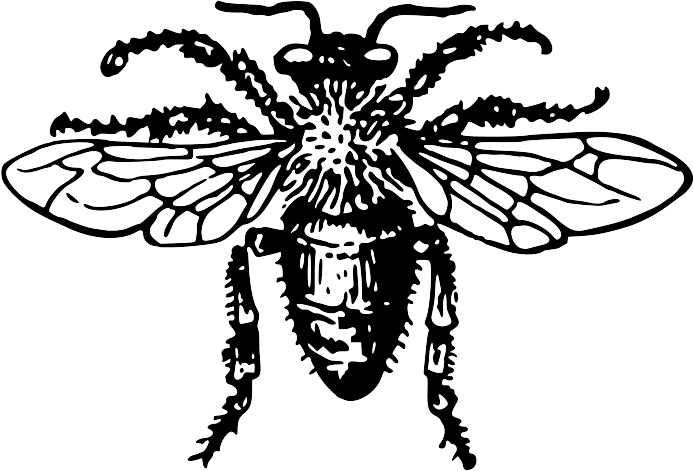**84 test images** | 0.984 | 0.982 | 0.992 | 0.991 | 0.966 | 0.988 | **0.987** | **1.3%** |
| **3 training images** | 0.841 | 0.890 | 0.954 | 0.940 | 0.746 | 0.917 | **0.919** | **8.1%** |
| **7 training images** | 0.946 | 0.926 | 0.973 | 0.965 | 0.816 | 0.950 | **0.951** | **4.9%** |
| **12 training images** | 0.947 | 0.942 | 0.975 | 0.977 | 0.882 | 0.958 | **0.960** | **4.0%** |
| **18 training images** | 0.960 | 0.947 | 0.979 | 0.981 | 0.875 | 0.963 | **0.965** | **3.5%** |
| **26 training images** | 0.968 | 0.953 | 0.982 | 0.984 | 0.906 | 0.968 | **0.970** | **3.0%** |
| **Uncropped data** | 0.924 | 0.898 | 0.956 | 0.941 | 0.775 | 0.924 | **0.928** | **7.2%** |
| **Automatic cropping** | 0.949 | 0.928 | 0.970 | 0.968 | 0.865 | 0.951 | **0.952** | **4.8%** |
| **Biomedisa interpolation** | 0.967 | 0.949 | 0.986 | 0.982 | 0.856 | 0.962 | **0.967** | **3.3%** |
| **AVIZO interpolation** | 0.925 | 0.925 | 0.915 | 0.914 | 0.848 | 0.946 | **0.928** | **7.2%** |
