# Supplementary material for: Natural variability in bee brain size and symmetry revealed by micro-CT imaging and deep learning
Source: PLoS Comput Biol. 2023 Oct 2;19(10):e1011529. doi: 10.1371/journal.pcbi.1011529 (PMC10569549; doi:10.1371/journal.pcbi.1011529)
Supplement: S2 Table — The reference score is based on Biomedisa’s standard configuration as used in the manuscript. Highlighted Dice scores indicate that the average Dice score is statistically significantly smaller than the average Dice score of the reference sample (t-test, p<0.05). Network size is represented by the number of filters up to the deepest block of the encoder part. The 110 three-dimensional honey bee images were divided into 26 training images, 30 three-dimensional validation images, and 54 three-dimensional test images. Similarly, the 77 three-dimensional bumblebee images were split into 13 three-dimensional training images, 20 three-dimensional validation images, and 24 three-dimensional test images. The final evaluation is conducted on the respective test images. (DOCX) [file pcbi.1011529.s014.docx]

**S2 Table. Effect of varying hyperparameters on segmentation accuracy.** The reference score is based on Biomedisa’s standard configuration as used in the manuscript. Highlighted Dice scores indicate that the average Dice score is statistically significantly smaller than the average Dice score of the reference sample (t-test, p<0.05). Network size is represented by the number of filters up to the deepest block of the encoder part. The 110 three-dimensional honey bee images were divided into 26 training images, 30 three-dimensional validation images, and 54 three-dimensional test images. Similarly, the 77 three-dimensional bumblebee images were split into 13 three-dimensional training images, 20 three-dimensional validation images, and 24 three-dimensional test images. The final evaluation is conducted on the respective test images.

| Batch size | 12 | 24 | 48 |
| --- | --- | --- | --- |
| Honey Bees | **0.9683 (p<0.001)** | 0.9703 | **0.9670 (p=0.027)** |
| Bumblebees | 0.9475 (p=0.948) | 0.9486 | **0.9405 (p=0.001)** |
| Combined | **0.9619 (p<0.001)** | 0.9637 | **0.9588 (p<0.001)** |
| Learning rate | 0.001 | 0.01 | 0.1 |
| Honey Bees | **0.9624 (p<0.001)** | 0.9703 | **0.9697 (p=0.027)** |
| Bumblebees | **0.9389 (p<0.001)** | 0.9486 | **0.9469 (p<0.005)** |
| Combined | **0.9551 (p<0.001)** | 0.9637 | **0.9627 (p<0.001)** |
| Network Size | 32-64-128-256 | 32-64-128-256-512 | 32-64-128-256-512-1024 |
| Honey Bees | **0.9656 (p<0.001)** | 0.9688 (p=0.112) | 0.9703 |
| Bumblebees | **0.9345 (p<0.001)** | 0.9486 (p=0.490) | 0.9486 |
| Combined | **0.9560 (p<0.001)** | 0.9625 (p=0.125) | 0.9637 |
