# Supplementary material for: Natural variability in bee brain size and symmetry revealed by micro-CT imaging and deep learning
Source: PLoS Comput Biol. 2023 Oct 2;19(10):e1011529. doi: 10.1371/journal.pcbi.1011529 (PMC10569549; doi:10.1371/journal.pcbi.1011529)
Supplement: S3 Table — Pearson correlation coefficient and p-value are given. Strong correlations (r>0.40) and significant correlations (p<0.05) are displayed in bold. Brain areas are labelled using the same abbreviations as in Fig 2. (DOCX) [file pcbi.1011529.s015.docx]

| **S3 Table. Correlation between absolute neuropil volumes (bottom left) and between relative neuropil volumes (top right) for honey bees (N=110).** Pearson correlation coefficient and p-value are given. Strong correlations (r>0.40) and significant correlations (p<0.05) are displayed in bold. Brain areas are labelled using the same abbreviations as in Fig 2. | | | | | | | |
| --- | --- | --- | --- | --- | --- | --- | --- |
| 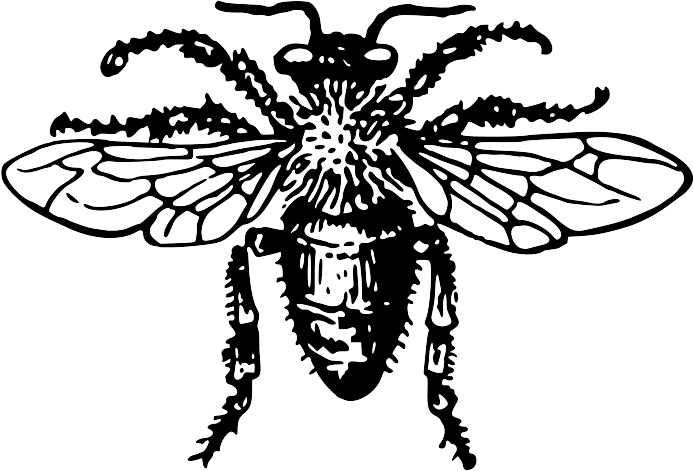 | **AL** | **MB** | **OL** | **ME** | **LO** | **CX** | **OTH** |
| **AL** |  | **-0.45**  **(p<0.001)** | 0.27  **(p=0.005)** | 0.24  **(p=0.010)** | 0.27  **(p=0.004)** | 0.08 (p=0.424) | -0.06  (p=0.511) |
| **MB** | 0.22  **(p=0.019)** |  | -0.26  **(p=0.006)** | -0.27  **(p=0.005)** | -0.18  (p=0.061) | -0.01  (p=0.901) | **-0.54**  **(p<0.001)** |
| **OL** | **0.71**  **(p<0.001)** | 0.35  **(p<0.001)** |  | **0.98**  **(p<0.001)** | **0.81**  **(p<0.001)** | 0.21  **(p=0.025)** | **-0.65**  **(p<0.001)** |
| **ME** | **0.69**  **(p<0.001)** | 0.33  **(p<0.001)** | **0.99**  **(p<0.001)** |  | **0.69**  **(p<0.001)** | 0.16  (p=0.088) | **-0.62**  **(p<0.001)** |
| **LO** | **0.69**  **(p<0.001)** | 0.39  **(p<0.001)** | **0.93**  **(p<0.001)** | **0.88**  **(p<0.001)** |  | 0.32  **(p<0.001)** | **-0.57**  **(p<0.001)** |
| **CX** | 0.32  **(p<0.001)** | 0.13  (p=0.168) | 0.35  **(p<0.001)** | 0.32  **(p<0.001)** | **0.43**  **(p<0.001)** |  | -0.21  **(p=0.025)** |
| **OTH** | **0.60**  **(p<0.001)** | 0.38  **(p<0.001)** | **0.59**  **(p<0.001)** | **0.57**  **(p<0.001)** | **0.57**  **(p<0.001)** | 0.30  **(p=0.002)** |  |
