# Supplementary material for: Natural variability in bee brain size and symmetry revealed by micro-CT imaging and deep learning
Source: PLoS Comput Biol. 2023 Oct 2;19(10):e1011529. doi: 10.1371/journal.pcbi.1011529 (PMC10569549; doi:10.1371/journal.pcbi.1011529)
Supplement: S5 Table — Mean (± standard deviation), minimal and maximal volumes (mm3). Global percentage of volume variation and inter-individual variability of brain and neuropils volume (%) within colonies (N = 77 bumblebees). Information is also given for the left and right sides of paired neuropils (i.e. AL, MB, OL, ME, LO). F-test, following LMMs, tests the significance of the fixed variable ”colony”, and results are displayed in bold when significant. Brain areas are labelled using the same abbreviations as in Fig 2. (DOCX) [file pcbi.1011529.s017.docx]

| **S5 Table. Total brain and neuropil volumes of bumblebees (N=77).** Mean (± standard deviation), minimal and maximal volumes (mm³). Global percentage of volume variation and inter-individual variability of brain and neuropils volume (%) within colonies (N=77 bumblebees). Information is also given for the left and right sides of paired neuropils (i.e. AL, MB, OL, ME, LO). F-test, following LMMs, tests the significance of the fixed variable ”colony”, and results are displayed in bold when significant. Brain areas are labelled using the same abbreviations as in Fig 2. | | | | | | | | | | |
| --- | --- | --- | --- | --- | --- | --- | --- | --- | --- | --- |
| **Neuropil** | **Mean ± s.d. (mm³)** | **Min (mm³)** | **Max (mm³)** | **%  variation** | **F-test** | **Relative volume (%)** | **Colony  A (N=18)** | **Colony**  **B (N=21)** | **Colony**  **C (N=21)** | **Colony  D (N=18)** |
| **Brain** | 0.862±0.127 | 0.520 | 1.089 | 52.27% | F(3,73) =1.854, p=0.145 |  | 42.53% | 43.08% | 51.65% | 29.79% |
| **AL** | 0.058±0.010 | 0.033 | 0.081 | 59.63% | F(3,73) =1.491, p=0.224 | 6.70±0.82 | 55.30% | 55.01% | 57.65% | 53.25% |
| ***Left*** | *0.029±0.005* | *0.016* | *0.041* | *61.90%* |  |  |  |  |  |  |
| ***Right*** | *0.029±0.006* | *0.014* | *0.040* | *64.33%* |  |  |  |  |  |  |
| **MB** | 0.266±0.041 | 0.141 | 0.340 | 58.70% | F(3,73) =2.004, p=0.121 | 30.91±2.43 | 49.47% | 41.35% | 58.70% | 36.19% |
| ***Left*** | *0.135±0.021* | *0.071* | *0.167* | *57.70%* |  |  |  |  |  |  |
| ***Right*** | *0.135±0.023* | *0.069* | *0.171* | *59.44%* |  |  |  |  |  |  |
| **OL** | 0.240±0.030 | 0.164 | 0.295 | 44.34% | F(3,73) =2.340, p=0.080 | 28.10±2.62 | 30.57% | 43.90% | 38.63% | 31.28% |
| ***Left*** | *0.119±0.016* | *0.072* | *0.146* | *50.38%* |  |  |  |  |  |  |
| ***Right*** | *0.122±0.015* | *0.082* | *0.150* | *44.83%* |  |  |  |  |  |  |
| **ME** | 0.182±0.023 | 0.123 | 0.229 | 46.37% | F(3,73) =2.014, p=0.119 | 21.25±2.17 | 32.26% | 46.37% | 37.47% | 31.38% |
| ***Left*** | *0.090±0.012* | *0.049* | *0.111* | *56.27%* |  |  |  |  |  |  |
| ***Right*** | *0.092±0.011* | *0.062* | *0.118* | *47.52%* |  |  |  |  |  |  |
| **LO** | 0.059±0.008 | 0.040 | 0.074 | 45.25% | F(3,73) =2.750, p=0.049 | 6.85±0.60 | 37.10% | 44.40% | 44.41% | 31.81% |
| ***Left*** | *0.029±0.004* | *0.020* | *0.038* | *47.20%* |  |  |  |  |  |  |
| ***Right*** | *0.030±0.004* | *0.021* | *0.042* | *50.59%* |  |  |  |  |  |  |
| **CX** | 0.004±0.001 | 0.001 | 0.007 | 85.13% | F(3,73) =0.727, p=0.539 | 0.47±0.11 | 75.33% | 75.30% | 85.13% | 66.70% |
| **OTH** | 0.294±0.065 | 0.087 | 0.408 | 78.65% | F(3,73) =0.919, p=0.436 | 33.82±4.49 | 55.55% | 59.48% | 78.65% | 40.37% |
