# Supplementary material for: Natural variability in bee brain size and symmetry revealed by micro-CT imaging and deep learning
Source: PLoS Comput Biol. 2023 Oct 2;19(10):e1011529. doi: 10.1371/journal.pcbi.1011529 (PMC10569549; doi:10.1371/journal.pcbi.1011529)
Supplement: S6 Table — Pearson correlation coefficient and p-value are given. Strong correlations (r>0.40) and significant correlations (p<0.05) are displayed in bold. Brain areas are labelled using the same abbreviations as in Fig 2. (DOCX) [file pcbi.1011529.s018.docx]

| **S6 Table. Correlation between absolute neuropil volumes (bottom left) and between relative neuropil volumes (top right) for bumblebees (N=77).** Pearson correlation coefficient and p-value are given. Strong correlations (r>0.40) and significant correlations (p<0.05) are displayed in bold. Brain areas are labelled using the same abbreviations as in Fig 2. | | | | | | | |
| --- | --- | --- | --- | --- | --- | --- | --- |
| 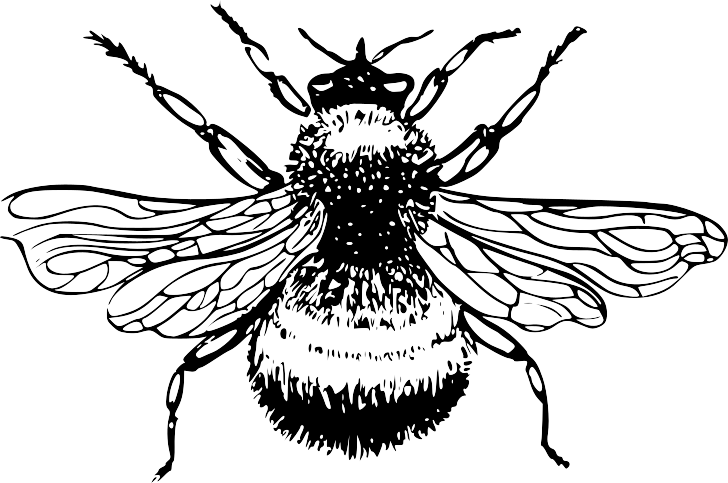 | **AL** | **MB** | **OL** | **ME** | **LO** | **CX** | **OTH** |
| **AL** |  | 0.28  **(p=0.015)** | **0.41**  **(p<0.001)** | 0.32  **(p=0.004)** | **0.60**  **(p<0.001)** | -0.03  (p=0.799) | **-0.57**  **(p<0.001)** |
| **MB** | **0.76**  **(p<0.001)** |  | 0.32  **(p=0.005)** | 0.30  **(p=0.008)** | 0.30  **(p=0.009)** | 0.07  (p=0.541) | **-0.78**  **(p<0.001)** |
| **OL** | **0.79**  **(p<0.001)** | **0.85**  **(p<0.001)** |  | **0.99**  **(p<0.001)** | **0.80**  **(p<0.001)** | -0.20  (p=0.078) | **-0.82**  **(p<0.001)** |
| **ME** | **0.74**  **(p<0.001)** | **0.82**  **(p<0.001)** | **0.99**  **(p<0.001)** |  | **0.69**  **(p<0.001)** | -0.18  (p=0.109) | **-0.79**  **(p<0.001)** |
| **LO** | **0.86**  **(p<0.001)** | **0.83**  **(p<0.001)** | **0.91**  **(p<0.001)** | **0.84**  **(p<0.001)** |  | -0.21  (p=0.061) | **-0.73**  **(p<0.001)** |
| **CX** | **0.49**  **(p<0.001)** | **0.52**  **(p<0.001)** | **0.50**  **(p<0.001)** | **0.48**  **(p<0.001)** | **0.49**  **(p<0.001)** |  | 0.06  (p=0.608) |
| **OTH** | **0.57**  **(p<0.001)** | **0.59**  **(p<0.001)** | **0.53**  **(p<0.001)** | **0.48**  **(p<0.001)** | **0.60**  **(p<0.001)** | **0.51**  **(p<0.001)** |  |
