# Supplementary material for: Natural variability in bee brain size and symmetry revealed by micro-CT imaging and deep learning
Source: PLoS Comput Biol. 2023 Oct 2;19(10):e1011529. doi: 10.1371/journal.pcbi.1011529 (PMC10569549; doi:10.1371/journal.pcbi.1011529)
Supplement: Attachment — Submitted filename: Response to Reviewers_v2.pdf [file pcbi.1011529.s019.pdf]

Response to Editors and Referees

PCOMPBIOL-D-23-00086
Natural variability in bee brain size and symmetry revealed by micro-CT imaging and deep learning

Dear Dr. Barbara Webb,
dear Dr. James O'Dwyer,
dear reviewers,

Thank you for considering our manuscript, "*Natural variability in bee brain size and symmetry revealed by micro-CT imaging and deep learning*," for publication in PLOS Computational Biology. We are grateful for the opportunity to submit a revised version of our work.

We appreciate the time and effort you and the reviewers took to provide valuable feedback and insightful comments on our study. The constructive input from the reviewers has been immensely helpful in refining our manuscript.

We are particularly thankful to the reviewers for acknowledging the merits of our method and finding our work of interest. Their positive comments, such as R1's recognition of the various merits of our work, R2's praise for the much-needed automated AI-guided approach for comparative brain analysis in evolutionary biology, and R3's recognition of the effectiveness of our method for insect brain volume segmentation, are truly motivating.

Based on the reviewers' recommendations, we have made significant improvements to our manuscript, incorporating a more detailed evaluation, including new Figures (S4−7 Figs) and S2 Table, and introducing S2 Fig to illustrate CT scans of Bumblebees with segmentation results, grund truth and errors. Furthermore, we have made all the data and code readily accessible in the official Biomedisa GitHub repository, as well as in the Supporting Information as S1 Appendix. For the underlying 3D label and image data, along with the trained neural networks, they can be accessed at https://biomedisa.org/gallery/. Please also note the following changes of the figures:

"mm^3" added to all axes with volumetric scale.
Fig 4: Symbol for light microscopy added.
Fig 7: Panels I and J removed, and new figures created for each (see Comment C3.8).
S1 Fig: Ground truth added (see Comment C1.13).

To aid in identifying the changes, we have clearly highlighted them within the manuscript, distinguishing ~~old text~~ that has been removed and new text that has been added. Please note that all line numbers mentioned in the responses refer to the revised manuscript including changes. We have also included a clean version of the revised manuscript for your convenience. In the following sections, we address each point raised by the reviewers meticulously.

We sincerely hope that these revisions demonstrate our commitment to enhancing our work and contribute positively to the PLOS Computational Biology Journal.

Thank you once again for this valuable opportunity.

Sincerely,

Philipp D. Lösel (on behalf of all co-authors)

Reviewer #1:

Reviewer Summary. The Manuscript "Natural variability in bee brain size and symmetry revealed by micro-CT imaging and deep learning" by Lösel et.al. is investigating the variations in total brain size of bees. To do so, the authors used semi-automated and fully automated methods to segment micro-CT scans of different bee specimens. Finally, the authors performed statistical analysis of the volumes of the bee brains and their inner parts.

It is a nice work which has various merits; however, the manuscript has some important issues that need to be revised before it can be considered for publication:

Reviewer Comment C1.1. In line 213, the authors claim that the dataset of their reference 29 is smaller though it is bigger. In Gronenberg -Couvillon publication the sample size of honeybees is 121.

Response. In reference 29, the total sample size of honey bees was indeed larger, but it was not homogeneous since it included samples taken from different subspecies i.e. 67 European and 54 Africanized honey bees, and the use of two bee strains may have led to biological differences in structural volumes, as discussed in lines 290−293. We have also clarified this point in lines 91−94 as follows:

"However, there is currently no clear connection between possible differences in brain neuro-architecture and behavioural variance due to the lack of data from comparative analyses (few studies [27] and sample sizes ranging from 7 [28] to 67 [29] individuals - [29] used 67 European honey bees and 54 Africanised honey bees)."

Reviewer Comment C1.2. The authors performed statistical analysis using 110 individuals of only one species of honeybees and 77 individuals of one species of bumblebees. Thus, it is a long stretch to generalize the results for all bee species, as there is a big variation between them. Therefore, I would suggest the authors to reconsider their title and be somewhat more precise/moderate.

Response. We agree that we cannot generalize our results on two model species (*Apis mellifera* and *Bombus terrestris*) to all bee species and made this clear in lines 497−501.

"Our results on two social bee species ~~honey bees and bumblebees~~ show high natural variability in brain area sizes that is stable across colonies~~, suggesting selection for variability supporting adaptive division of labour~~. These anatomical differences could potentially generate behavioural variability and support adaptive collective behaviour, thereby calling for future studies in more species with contrasted social ecologies."

However, we would like to keep using "bees" in the title instead of "Western honey bees and buff-tailed bumblebees" in order to keep it short.

Reviewer Comment C1.3. It is unclear whether the authors considered the age and the body size of the individuals as a factor during their statistical analysis. They mention that "… workers vary little in body size" but did they compare the body sizes of their dataset? Was the head size, eyes, or antenna size measured and compared with the associated neuropils? Also, was the age of all individuals taken as a parameter in the linear mixed model? Finally, I would expect logarithmic transformation of the comparative data, as ecological data are usually skewed to the right.

Response. Unfortunately, we did not have age and body size information and therefore could not add them as a factor in the statistical analyses. This was now clarified in lines 526−528:

"The analysed honey bees and bumblebees were of unknown age, and no body size information was available for these specimens."

We did not apply logarithmic transformation of our data because they were normally distributed as now mentioned in line 544 in the Methods section "Statistical analysis":

"Brain volumes were normally distributed."

Reviewer Comment C1.4. As we can see in table S3, the number of bees from each hive was quite different. We can see that even within this small size of hive sample, the variability is not trivial, especially for OTH. As the number of individuals in each hive sample is getting bigger so does the variability in brain volume. Thus, how can the authors generalize their results for all honeybees? How did the authors choose the fixed and random factors? Were the specific hives representatives of different features in honeybees? Was the population treated as numerical factor and if not, what was its threshold?

Response. Thank you for this comment. The level of variability within hives is indeed quite complex to interpret. As mentioned in the methods section "Sample preparation and CT scan" all our honey bees were from the same strain but sampled in two different apiaries. We have no information about the age of the bees nor their exact foraging experience or on potential external environmental stressors in the study area. Although our sample size is larger than in previous studies, we think that we do not have enough data and knowledge about our populations to fully understand the origin of this variability. We therefore prefer to remain descriptive and we have removed previous attempts to interpret this variability in evolutionary terms (see our response to Comment C3.3 by reviewer 3) or to generalize to all bee species (see our response to your Comment C1.2).

Reviewer Comment C1.5. The authors describe the statistical analysis of the variations within colonies but not the analysis between colonies.

Response. The statistical analysis between colonies is described in lines 545−548 which reads:

"To analyse the inter-colonial variations of brain volume, we conducted linear mixed models (LMMs) using the lme4 package [68~~6~~], with hive as fixed effect and population as random factor. LMMs were followed by F-tests to test the significance of fixed categorical variables using the anova function in the car package [69~~7~~]."

See also lines 362−364:

"In our study, h~~H~~oney bees in the nine colonies exhibited overall similar average brain volumes (F-test: p=0.428; Fig 6A and Table 1), hence, inter-colony differences do not explain the substantial (32%) inter-individual variability."

Reviewer Comment C1.6. It is not very clear when the authors talk about sets of images, 3D images, 2D images, or scans; e.g., in line 134 they talk about different numbers of images, in line 141 about scans, in line 163 about 3D images. Even though they are all the same, it is better to keep one description for the readers' benefit.

Response. Thank you for your comment. To enhance clarity, we now use the terms "3D image", or "three-dimensional image" when it follows a numeric value, throughout the manuscript. In rare cases where we refer to a slice (i.e., a 2D image) extracted from a 3D image, we now explicitly indicate it.

Reviewer Comment C1.7. The authors mention in line 178 that their automatic

segmentation is highly accurate, which is too subjective a statement. 12.5% of an error greater than 4% is higher than the 9.4% with very few errors in bumblebees. That, I believe, comes as a result of an overfitted network due to the lack of training data. I would suggest the authors used transfer learning algorithms to retain the weights from honeybees and refine them for the bumblebees. In that way their network will be more generalized.

Response. Thank you for this important comment. We have weakened the statement of the headline (lines 218−219) as follows:

"Accurate aAutomatic segmentation of bee brains with noticeable performance variations in bumblebees ~~is highly accurate~~"

While the segmentation accuracy is indeed lower in bumblebees compared to honeybees, it is important to note that this discrepancy arises from the lower image quality of the bumblebee data, rather than overfitting. To illustrate this, we have included S2 Fig, which showcases the segmentation results and errors of specific bumblebee images.

Regarding the size of training data, it is important to consider that we utilize 3D patches extracted from the 3D volumes, resulting in a substantial number of training patches. Each 3D image, scaled to 256x256x256 voxels, yields 343 patches of 64x64x64 voxels based on a stride size of 32 voxels. For the 13 volumetric training images of bumblebee brains, this amounts to a total of 4,459 individual training patches. These 3D patches are then utilized to train the neural network.

During inference, overlapping 3D patches of 64x64x64 voxels are employed to generate the 3D segmentation result. Both the standard accuracy and the Dice score stay close to their maximums (maximum standard accuracy after 154 epochs for bumblebees and after 197 epochs for honey bees, maximum Dice score after 129 epochs for bumblebees and after 123 epochs for honey bees) even up to 200 epochs (S4−7 Figs), indicating that overfitting is not a concern. Although, it is true that the loss function slightly starts to increase from around 100 epochs (S5 Fig). It is important to note that we always used Biomedisa's standard configuration to demonstrate what users can expect without optimizing parameters to their specific dataset.

While transfer learning is particularly valuable when training data is limited, for the bumblebee dataset we conducted a comparison between fine-tuning the network trained on the honeybee dataset, following Amiri et al. 2020 (keeping the decoder part fixed and only training the encoder part), and training from scratch using different numbers of training images (S3 Fig). As expected, fine-tuning was better when the number of training images was small (3 to 7 training images) but not when more than 12 training images were utilized. It is worth noting that despite the similarity in brain shape, the bumblebee CT scans differ significantly from honeybee images. In particular in terms of contrast between brain areas, especially concerning CX (S2 Fig).

Reviewer Comment C1.8. It is unclear why the authors chose 200 training epochs in such a small sample.

Response. The sample size, as mentioned earlier, is not small. Although the accuracy remains mostly unaffected, employing 200 epochs might prove inefficient. The decision to use 200 epochs was made to ensure thorough training of the network as now mentioned in lines 270−271. However, Biomedisa provides the option of early stopping to prevent unnecessarily prolonged training.

Reviewer Comment C1.9. Also, why the variation set is larger than the training set? How did the authors choose to split their data?

Response. The decision to have a larger validation set compared to the training set was made to align with the process used in analyzing our bee brains and what a typical Biomedisa user would do. We added the following paragraph to the "Increasing the number of 3D training images" section (lines 606−611):

"*While the conventional practice often involves allocating approximately 80% of the data for training and 20% for testing, or using a split of 60% for training and 20% each for validation and testing, we opted for a training dataset size that aligns with the analysis procedure for our specific bee brain dataset. Thus, we used up to 26 CT scans for training, selecting a similar amount for validation, considering the compute-intensive nature of validation during training, and assigned the remaining 3D images for testing.*"

Reviewer Comment C1.10. It is common to choose the values of hyperparameters based on the accuracy and performance of the network during training and validation. Can the authors provide such data?

Response. Biomedisa follows the idea of a simple and out-of-the-box tool. Therefore we chose the standard configuration of Biomedisa as described in the manuscript. They have proven to perform well on diverse image datasets. Although researchers can adapt the parameters to their own needs, we wanted to demonstrate the process based on the standard configuration without optimization making it simple to use. Nevertheless we checked the accuracy for varying learning rate, batch size, and network size and included this information in the new S2 Table. The accuracy for varying epochs can now be found in S4 & S6 Figs, which have been added to the Supporting Information and discussed in detail in the section "Accurate automatic segmentation of bee brains with noticeable performance variations in bumblebees". Most interestingly, choosing a smaller network size (up to 5 blocks with a maximum of 512 channels for the deepest layer) does not give statistically significant worse results but reduces computation time from 13 to 10 hours using 4 NVIDIA V100.

Reviewer Comment C1.11. How was the loss changing during training?

Response. The loss for the bumblebee dataset can be found in S5 Fig and the loss for the honey bee dataset in S7 Fig. The minima are after 34 epochs and 62 epochs, respectively.

Reviewer Comment C1.12. An ROC curve is essential to check the overfitting or underfitting of the network.

Response. The Receiver Operating Characteristic (ROC) curve is not typically used for measuring accuracy in image segmentation tasks. The ROC curve is commonly employed in binary classification problems where the goal is to distinguish between two classes, such as detecting whether an image contains a specific object or not. Image segmentation, on the other hand, involves dividing an image into meaningful regions or segments. Accuracy in image segmentation is typically evaluated using metrics that assess the quality of the segmentation masks, such as Intersection over Union (IoU), also known as the Jaccard index, or the Dice coefficient. These metrics measure the overlap between the predicted segmentation and the ground truth segmentation. The ROC curve is not directly applicable to segmentation because it requires a binary classification output. In image segmentation, the output is a pixel-wise mask or a set of regions, rather than a binary classification label. To evaluate the performance of an image segmentation model, it is more appropriate to use metrics like IoU, Dice coefficient, or pixel-wise accuracy, which focus on the quality of the segmentation itself rather than classifying individual pixels as positive or negative.

The basic problem of applying ROC to image segmentation is that you take into account the background but you always have a huge amount of background labeled correctly resulting in

AUC often of 1.0. In contrast, the Dice score does not consider the background but the overlap of a brain area with its ground truth making it much more reliable.

Reviewer Comment C1.13. In S1 Fig. it is unclear whether the network overpredicted the areas or mislabelled them. It would be much better if the authors provided the ground truth image too.

Response. We added the outline of the ground truth data to the right column of S1 Fig and added a similar figure for the bumblebee dataset (S2 Fig). While there is some overprediction, the errors occurring are most often mislabelling.

Reviewer Conclusion. Based on all these points, I think the manuscript needs to be significantly revised to warrant publication in PLOS Computational Biology.

Reviewer #2:

Reviewer Summary. The authors report on automated brain image analysis that facilitates large-scale analysis of neuropils and their variability in brain architecture in social Hymenoptera. By choosing social insects that exhibit elaborated behavior, such as honey- and bumble bees the authors report on lateralization of brain structures thus partly confirming partly previous reports on lateralization in bumble bees. An automated AI guided approach, as reported here using the open-source platform Biomedisa is very much needed in comparative brain analysis in evolutionary biology. The usage of the Biomedisa's smart interpolation is already in public use. The novelty of the current manuscript is, as to my knowledge, its extension of the AI guided automation for analysis a large number of specimens.
The contrast in the CT scans is of sufficient quality to provide a clear separation of soma layers vs. neuropil, and neuropil vs neuropil, though the resolution (5.4 isotropic voxel size) is quite low compared to high-resolution CT or standard confocal imaging. (See my comments below).
In general, I find the approach promising from its technical side, and therefore potentially recommend their publication in PLOS Biology.

Reviewer Comment C2.1. The results should be discussed more profound and compared to other approaches in its efficiency to facilitate segmentation of brain neuropils (see below). I regard this important knowledge for researchers prior to advance to large-scale scanning of specimen, i.e., there is a minimum of about 20 samples, that researcher have to sample for their experiments as a training set for the AI .
-Is the CT methods as efficient as a neuropil- specific marker compared to antibody based synapsin or fluorescent marker such as Lucifer yellow.

For example
- are other histological procedures (neuropil marker Synapsin, Lucifer Yellow) and scanning times less time consuming and giving better contrast (thus decreasing the number of training images for optimal segmentation) and might be better suited for AI guided segmentation?

Response. It is true that our protocol for preparing the samples is rather long (15 days of incubation), and that other methods may help gaining time, although processing brains for synapsin staining typically takes at least around a week, see e.g. Kraft *et al*. Cell Tissue Research (2023). We did not test such staining methods, which are indeed useful to unravel possible differences in neuropil volumes, but we believe that they can be complementary, and we agree that improving the contrast might help to optimize the segmentation. We added a sentence in our conclusion (lines 493−495), in a way to encourage other

researchers using such markers to test our automatic quantification tools:

"Additionally, this method can complement other histological procedures commonly used to study neuropiles, such as immunostaining and Lucifer yellow, by potentially reducing the number of required training images through improved contrast."

Reviewer Comment C2.2. - Muratore (2022) using synapsin stained brain tissue (Muratore IB, Fandozzi EM, Traniello JFA. Behavioral performance and division of labor influence brain mosaicism in the leafcutter ant Atta cephalotes. J Comp Physiol A. 2022;208(2):325-44.) The authors might want to cite and discuss their segmentation strategy. Synapsin antibodies are most commonly used neuropil marker in insects, giving high contrast. It would be interesting to see of how efficient the AI guided segmentation tools of BioMedisa is compared to the Muratore study.

Response. As mentioned in the response to Comment C2.1, we are convinced that using techniques to increase the contrast will positively affect segmentation accuracy and thus help to reduce the amount of required 3D training images. We acknowledge that other histological procedures, like synapsin staining, can offer high contrast and may be advantageous for specific investigations into neuropil volumes. We believe that future studies could investigate and publish on the comparative performance of various neuropil markers and imaging approaches using Biomedisa for specific research goals, and we mentioned the study Muratore (2022) in our conclusion (lines 495−497) as follows:

"Future investigations could explore and publish on the comparative performance of various neuropil markers and imaging techniques, including confocal microscopy [61]."

Reviewer Comment C2.3. - A simple, fast histology was employed in honeybees using Lucifer Yellow as a neuropil marker by Rybak 2010 (Rybak J, Kuss A, Lamecker H, Zachow S, Hege HC, Lienhard M, et al. The Digital Bee Brain: Integrating and Managing Neurons in a Common 3D Reference System. Front Syst Neurosci. 2010;4:30.). An statistical shape model composed out of 20 training images was then used for automated segmentation. In this respect: In your approach - does the pre-segmentation need to be done for each specimen over and over again. Or does the AI builds a a memory of relative position and /or shape of neuropiles during the training procedures?

Response. Our presented workflow is as follows: Use the semi-automated segmentation (Biomedisa interpolation) to create 3 training images and train the first neural network. Use this network to automatically segment the next 4 bee brains followed by manual correction. Then, use all 7 images to train the next neural network and use this network to segment the next 5 bee brains followed by manual correction. Repeating this process, we trained networks based on 3, 7, 12, 18, and finally 26 images. Consequently, the AI successively builds on previously acquired knowledge during the training procedures and it is not necessary to start the creation of each training image from scratch.

Reviewer Comment C2.4. - what exactly means: cropping of the data during segmentation ?

Response. We consider two scenarios. First, the 3D CT scan of the entire bee head, and second, the 3D CT scan cropped to the region of interest, i.e. the bee brain. As discussed in the "Cropping ~~Uncropped~~ 3D image data to the region of interest improves accuracy" subsection of the Methods section (lines 625−635), the segmentation accuracy is higher when considering cropped image data. To avoid manual cropping, we also present how one can use Biomedisa to train an additional neural network ("Automatic cropping"), which performs an automatic cropping of the image data to the bee brain before the actual segmentation of the bee brain.

Reviewer Comment C2.5. - In your segmented data sets the color of paired neuropils appear the same color for left and brain hemispheres. How does you, the AI indeed distinguish left/right ?

Response. Indeed, the neural network does not distinguish left and right sides. Although it is possible to label the left and right side of each paired neuropil with different labels and train the network in Biomedisa to automatically segment them separately, we haven't tested that. Here, we segment the paired neuropils as one object, and separate left and right side in an automatised post-processing step (see also our response to your Comment C2.12). The script is included in the Biomedisa Git repository as now mentioned in the Methods section "Statistical analysis".

Reviewer Comment C2.6. - in this respect: Automatic segmentation: how does the AI knows the identity of the neuropil 3D objects, or was the naming done manually?

Response. The semi-automated segmentation of the first 3 training images was performed using the Biomedisa Interpolation in combination with Amira. In Amira, the different neuropils were labeled each with a unique value and named manually by the user. Both the Biomedisa interpolation and the Biomedisa AI preserve label values and names by transferring the Amira header to the result as now mentioned in lines 175−176 in the "Automatic segmentation of bee brains considerably speeds up analysis" section as follows:

"*This integration ensures the preservation of label values and names, streamlining the exchange of data and facilitating seamless collaboration between platforms.*"

Reviewer Comment C2.7. - Comparing the volume data of this CT study (Table 1) with Brandt (2005: Table 2) that uses synapsin histology but the same definition of neuropils (except that for the CB the PB and Noduli of the CX were omitted in Brandt (2005): the absolute volume of all neuropils is app. 70-75 % smaller compared to the CT volume of this study, with exception of the MB neuropil this 58 % smaller. The reason for that might the histological procedure (more shrinkage in synapsin histology. In relative numbers (% neuropil/total brain) the MB differ by 5 % (CT = 25 %, confocal synapsin = 20 %).

Response. You are right, these differences may be due to differences in tissue processing methods, and thus in the extent of neuropil shrinking, between studies. We acknowledged this possible factor of variation in the new version of the manuscript (lines 282−284):

"*As histological procedures differ across studies, we cannot exclude that variations in the extent of tissue shrinkage may also contribute to differences across datasets.*"

Reviewer Comment C2.8. - Although it is not in the focus of your study, I suggest to add a column with the relative volumes given as percentage in Table 1. That will allow an easier comparison with previous and future studies. I suggest to mark and separate the OL in Table 1 / Figure 5 as it is the sum of ME and LO.

Response. Thank you for your remark. We added the definition of OL as the sum of ME and LO in Table 1 and Figure 5. We also added relative volume (mean±s.d.), expressed in percentage, in Table 1.

Reviewer Comment C2.9. - Brain = AL +MB +ME +LO +CX +OTH ?

Response. Indeed, you are right. Line 134f. now reads:

"*Total brain volume was calculated as the sum of AL, MB, ME, LO, CX and OTH.*"

Reviewer Comment C2.10. - In Figure 5 n=59 for MB ?

Response. In Figure 5, N=110 for MB as well. While separating right and left was challenging for some individuals, we however managed to retrieve the volume of the whole neuropil for all honey bees.

Reviewer Comment C2.11. - All raw data of all specimen should be provided in a supplementary excel tables.

Response. Thank you for your remark, raw data was added to the Biomedisa GitHub repository as now mentioned in the the Data Availability Statement as follows. Additionally, it was included in the Supplementary Information as S1 Appendix:

"*Specifically, the evaluation and R script, along with the volumetric measurements, required for replication and analysis are also available in this GitHub repository.*"

Reviewer Comment C2.12. - Though the quality of automated segmentation is good, I am puzzled by the high amount of fused MBs in the samples, about the half of the Apis specimen. What could the reason? The most distal medial lobes tend to merge into each other. May be an sub-script can be programmed to create a automated separation?

Thank you for your comment. As described in response to Comment C2.5, the left and right sides were not individually segmented as distinct objects. While AL, ME, and LO could be easily separated using a post-processing script, the proximity and contact of the left and right sides of MB posed a challenge in their separation after segmentation. Although it is possible to train the network to label left and right sides individually, this would require the left and right sides to be distinguished in the training data. As we view this as a valid next step, we have now added the following sentence to the section "Optic lobes showed left-right asymmetry in bumblebees but not in honey bees" (lines 433−438) to address this aspect:

"*The proximity of left and right sides in MB structures posed difficulties in their post-segmentation separation, resulting in a comparison based on only 59 honey bees and 36 bumblebees. However, there is potential for improvement in the future by considering a separation of the left and right MB structures in the training data itself.* ~~*Note that left and right MB structures were often difficult to separate, so the comparison of the two MB sides was only based on 59 honeybees and 36 bumblebees.*~~"

Reviewer Comment C2.13. - A clear separation from connective tracts such as the lobula-to-optic tubercle tract seems to be difficult in CT imaging and these tracts are partly reconstructed (as seen in the reconstruction of Figure 2E). Where this tract belongs to? the lobula or the OHT (protocerebral lobe)?

Response. You are right, and actually this difficulty might also contribute to explain volume differences between this and other reconstruction approaches. This has been acknowledged in lines 284−286:

"*In addition, CT imaging does not always allow a clear separation between connective tracts and neuropiles, which may lead to a slight over-estimation of some neuropil volumes (without any expected impact on inter-individual comparisons).*"

As for the precise case of the prominent anterior optic tract, it was included in the "OTH" volume as mentioned in line 132.

Reviewer Comment C2.14. - Optical tubercle: in your CT scans the optic tubercle was very well distinguishable. Why not take the volume data in your list. The optic tubercle is one of

the main outputs of the OL to the protocerebral lobe.

Response. You are right, but in this first approach we preferred to focus on the main neuropils which are classically studied. Since the optic tubercle was specifically outlined and measured in only 2 out of the 11 previous studies, and only on honey bees, we did not consider this (and other) brain structures in our analysis, for the purpose of comparing volume values with previous studies. However we will definitely integrate the optic tubercle in our follow up study that is in preparation.

Reviewer Comment C2.15. - line 31 : 110 honeybees and 77 bumble bees ?

Response. The exact number per species is given in the introduction (line 95f.) which reads:

"By collecting 3D imagevolumetric data from theon brains of 110 honey bees (Apis mellifera) and 77 bumblebees (Bombus terrestris) [...]"

Reviewer Comment C2.16. - are all species foragers as in the honeybees ? if so variations might be due to foraging experience, too ?

Response. Indeed, for bumblebees, we did not control the age nor experience, as now mentioned in the methods (lines 526−529):

"The analysed honey bees and bumblebees analysed were of unknown age. While all honey bees were strictly foragers, bumblebees were randomly collected and may therefore constitute a more heterogeneous population."

and in the results (lines 396−398):

"Note, however, that the 77 bumblebees randomly sampled in the four source colonies were likely to be more heterogeneous in terms of behaviour than the 110 honey bees, which were all only foragers."

Reviewer Comment C2.17. - line 73: bees and some and ants beetles are model species

Response. You are right. We deleted "non-model" in the following sentences of the introduction.

"Recent improvements in resolution and acquisition times [8,9] have broadened the application of micro-CT to a wide range of animals, including non-model various species of small-sized insects […]"

"Here we demonstrate how deep learning can significantly speed up 3D image analysis for large-scale comparative brain studies in insects non-model species by using the semi-automated […]"

Reviewer Comment C2.18. - line 139: in the remaining 84 CTs you did not perform manual corrections on selected pre-labeled slice ? Or, how are these checked for quality ?

Response. All segmentation results, whether obtained through semi-automatic or fully automatic methods, undergo manual verification and correction. Then, the performance of the semi-automatic segmentation is evaluated by comparing the results with the corrected versions of the 26 three-dimensional training images (S1 Table). Similarly, the performance of the automatic segmentation is assessed by comparing the results with the corrected versions of the 84 three-dimensional test images (S1 Table). Our focus was to demonstrate the level of effort required by users to correct the segmentation results accurately.

Reviewer Comment C2.19. - In bumble bee, see below, line 152 you did not perform any quality check ?

Response. We assessed the performance of the automatic segmentation on the 64 bumblebee test brains by comparing the manually corrected segmentation results with those obtained from the neural network (lines 238−241 and S1 Table). Unlike the honeybee data, we originally did not conduct a performance test based on the number of training images for the bumblebee dataset. Instead, we chose to use 13 training images based on the results obtained from the honeybee image test. However, we have now included a performance test for the bumblebee dataset with an increasing number of 3D training images in S3 Fig and mentioned that in lines 621−623 as follows:

"Comparable results are achieved by utilising 3 to 26 three-dimensional training images from the bumblebee dataset, accompanied by 20 three-dimensional validation, and 24 three-dimensional test images (S3 Fig)."

Reviewer Comment C2.20. - line 174-176: it would be useful to give the average time / one brain for segmentation times

Response. The time required for segmenting a single brain is specified within the same paragraph: 5−10 minutes for automatic segmentation, 2−3 hours for semi-automatic segmentation, and 5−6 hours for conventional manual segmentation. A detailed explanation of the overall time calculation can be found in the "Manual processing times with or without Biomedisa" subsection of the Methods section.

Reviewer Comment C2.21. - also it should be mentioned that the automated, and linear segmentation of Amira segment Tools, including water-shed, considerably reduce the "pure" time of manual segment each slice. Depending on the contrast, simple threshold segmentation sometimes is sufficient to segment neuropils very fast… In other words: what is "purely" manual segmentation, indeed ?

Response. It is true that the Amira segmentation tools considerably speed up the segmentation process compared to segmenting each slice manually. However, our reference time for conventional segmentation in line 188 is based on using Amira segmentation tools such as linear interpolation and watershed. We clarified what we mean with conventional segmentation in lines 180−182 as follows and replaced *"*purely manual segmentation" by *"*conventional manual segmentation*"* throughout the manuscript:

"For both datasets, this automatic segmentation considerably reduced the time and effort required for 3D image analysis compared to conventional~~purely~~ manual segmentation (manually segmenting individual slices, followed by linear interpolation and manual correction)."

Reviewer Comment C2.22. - line 232: study by Gronenberg(2010) : lightmicroscopy but not electronmicroscopy !! They do compare 67 european vs 54 africanized honeybees.

Response. Thank you for your remark, we updated Figure 4 accordingly.

Reviewer Comment C2.23. - line 233: relative volume: are your data different from Gronenberg (2010) ? discussion

Response. The two datasets are mostly coherent regarding the analysis of relative volume (see lines 309−310).

"Again, we found strong correlations between the relative volumes of OL, LO and ME,

consistent with previous studies [29].”

Reviewer Comment C2.24. - line 241: you already reported relative volume in line 233 ? your statements are confusing here.

Response. Thank you for bringing attention to potential confusion in the text. To clarify, we first examined the correlations between the absolute volumes of individual neuropils and the total brain volume (lines 295−300). Subsequently, we analyzed the correlations between the relative volumes of individual neuropils and the total brain volume (lines 300−303). In the following section, we investigated the correlation between the absolute volumes of all individual neuropils (lines 304−308) and then examined the correlation of relative volumes among all individual neuropils (lines 308−317).

Reviewer Comment C2.25. - line 762 ASSD?

Response. Thank you. The abbreviation was clarified in the figure text.

Reviewer Comment C2.26. - line 765 un-copped ?

Response. Thank you. It was corrected to auto-cropping.

Reviewer Comment C2.27. - Figure1
- on the left side the time is noted for one specimen, but on the right side an assembly of brains are shown. Times for manual correction should be noted accordingly.

Response. Thank you for your comment. You are correct, however, including the total amount required for all brains can make the figure more complex. The loop in the box represents the training of 3, 7, 12, 18, and 26 brains, with varying numbers of segmented and manually corrected images (4, 5, 6, and 8 respectively) in each step. Prior to exiting the box, the remaining 84 brains were segmented and manually corrected. We added the following sentences to the caption of the figure to provide more precise information in this regard:

“For the honey bee dataset, the loop within the white box represents the training process for 3, 7, 12, 18, and 26 brains. Each step involved a different number of segmented and manually corrected 3D images, specifically 4, 5, 6, and 8 respectively. Upon completing the loop, the remaining 84 brains were segmented and manually corrected. All processing times provided are average times per brain.”

Reviewer Comment C2.28. - Are the manual corrections always done on selected slices? or the whole volumen ?

Response. Manual correction involved importing the complete 3D result and original 3D image data into AVIZO for manual adjustments as needed. In instances where the result exhibited significant flaws, the segmentation was redone from scratch using either AVIZO's linear interpolation or Biomedisa's smart interpolation.

Reviewer Comment C2.29. - Does the ‘smart Biomedisa interpolation’ include more sophisticated algorithm than e.g. the water-shed interpolation of Amira ?

Response. Indeed, the smart interpolation method utilizes weighted random walks that start from the pre-segmented slices and propagate throughout the volume. By assessing the number of hits of a voxel, one can assign the voxel to the background or any of the labels. The weighting is based on the image data, enabling the random walk to remain within areas similar to its starting region. This technique differs from algorithms like the traditional random

walker or graph cut, as it eliminates the need for manual parameter configuration. It was also specifically developed for large 3D image data and calculation on GPUs. For further details, please refer to reference 19 in the manuscript (Lösel 2016).

Reviewer Comment C2.30. - Figure 3
- line 765 correct auto-copping

Response. Thank you for your comment. It was corrected to auto-cropping.

Reviewer Comment C2.31. - Figure 4 revise
- the figure is difficult to read and confusing: Haddad (35) deep blue and then again purple ?

Response. In their study, Haddad et al. used both nuclear magnetic resonance microscopy and confocal microscopy, that is why this study appears in both color codes.

Reviewer Comment C2.32. - volume in mm^3 for the y-axis ? provide the raw data

Response. Yes, all volumes are expressed in mm^3 as indicated in the figure caption. The volume units are now displayed along the graph axes. Raw data was added to the Biomedisa GitHub repository and added to the Supporting Information (S1 Appendix).

Reviewer Comment C2.33. - the black circles (CT-scan)I can't see it in the graph ?

Response. CT scan was only used in one study (Greco and Stait-Gardner) from which we were only able to retrieve AL volume (*coral*).

Reviewer Comment C2.34. Figure 6
- what is LMM

Response. The statistical analysis LMM is described in line 546 which reads:

"[...] we conducted linear mixed models (LMMs) using the lme4 package [68~~6~~]".

We added it in the list of abbreviations used in the manuscript (line 886).

Reviewer Comment C2.35. - are inter individual variations within each have shown here ? and there is not variations between the two hives ? Indicate and explain.

Response. This figure shows the variation of absolute volumes between hives (H1 to H9 - x axis). Boxplots show the variation of volumes within individuals of the same hive. We added the following boxplot description to the caption of Figure 6:

"Boxplots show median volumes (intermediate line) and quartiles (upper and lower lines)."

We did not investigate in this paper the differences between populations (6 hives in purple *vs.* 3 hives in green), which was only used as a random factor in the LMMs (see line 547).

Reviewer Comment C2.36. - Figure 7
- mm^3 for the y-axis ?

Response. The volume units are now displayed along the graph axes.

Reviewer Comment C2.37. - why are the mean from other CT studies so different ? Are they ?

Response. We only included two other CT studies to compare with, which indeed have quite

different values. This may be due in part to the fact that the values from Smith et al. (2016) were obtained from a combination of workers and males (while we used only workers). Another difference probably arises from age, as they mention that their “sampled brains were not a mature adult size given we sampled very young bees (only four days old) with little experience and that brain structure volumes have been reported to increase with age by as much as 37%.” As for the other study by Rother et al (2021), it was based on a subset of the Smith dataset and was thus removed from the comparison, as suggested by Reviewer 3 (see our answer to Comment C3.8).

Reviewer Comment C2.38. - line 782-783 I do not understand the sentence. In Brandt (2005) and Haddad the volume were measured in order to eliminate all variations that are due to segmentation. In contrast: other studies were done to measure natural variability ….

Response. Thank you for your comment. We have revised the statement to provide clarity on why we did not include other data to Fig 4A as follows:

“For the total brain volume, comparisons with other studies are not shown ~~because of the cumulative uncertainties of the measurements~~ some studies did not provide total brain volume information, while others measured different neuropils compared to our study.”

Reviewer Comment C2.39. - The authors provide volume in cubic millimeter. In insects volume measures are mostly in cubic-micrometer (scientific format). I took the time to convert and compare the data from this study with the data from Brandt (2005). To fascilitate with most other insect studies can you provide an excel tables with your numbers and properly convert ?

Response. Volumes of neuropils in both mm^3 and μm^3 are now provided in the raw data.

Reviewer Comment C2.40. - The mismatch might be due to a different definition of neuropil borders and / or a different contrast in CT versus confocal synapsin staining. For example, the tracts between medulla and lobula are difficult to differentiate.

Response. You are right, we now acknowledge this potential factor of variability in the manuscript (see our response to your Comment C2.1).

Reviewer Comment C2.41. - A final comment: How much of the reported variability is due to natural variability, and how much contribute the technical and segmentation procedures to the variance of your data?

Response. This is an excellent question. Unfortunately, we do not have the data to tease apart these two possibilities. For this, we would need to design and run new experiments. For instance one could repeat the current study several times and vary technical parameters (e.g. staining procedure, segmentation) and characteristics of the bee population (genetic strains, patrilines etc.) one by one to reveal their effects on variability.

Reviewer #3:

Reviewer Summary. In this paper, the authors present a deep learning method for analysis of 3D volumes. The method is applied to volumes of bee brains to demonstrate that it can effectively identify and segment the volumes of different brain areas. The authors then perform some comparative analyses of the data and make some inferences about how this relates to inter-individual and inter-species differences.

In general, I think that this method seems like an effective way to segment insect brain volumes and the paper is well-written and clear. I do think that the paper is sufficiently rigorous and of general interest to warrant publication. I have some relatively minor comments however that I think should be addressed to improve clarity.

Reviewer Comment C3.1. Line 90: please provide the species here.

Response. We changed the sentence in lines 95−96 as follows:

"By collecting 3D image~~volumetric~~ data from the~~on~~ brains of 110 honey bees (Apis mellifera) and 77 bumblebees (Bombus terrestris) [...]"

Reviewer Comment C3.2. Line 134 (and, for example, line 455): it is confusing to call the image stacks for 3D volumes 'images' and this could lead to misunderstandings. Throughout the text, please be clear when referring to 'volumes' and when referring to images (i.e. 2D images).

Response. Thank you for your comment. We clarified throughout the manuscript (see our response to Comment C1.6 by reviewer 1).

Reviewer Comment C3.3. Line 285: I do not understand how this statement follows logically from the previous one. I do not think that any evidence is presented to suggest that there are adaptive levels of brain size variation and I therefore think that this sentence should be removed. At no point were divisions of labour taken into account. All honey bees were foragers and the bumblebees were selected randomly and therefore their roles were unknown. While there is some evidence of a trend for smaller bumblebees to take on tasks in the hive, while larger ones are foragers, this is not at all clear and differs from hive to hive.

Response. Thank you for this important comment. We entirely agree and we have removed the following statement (lines 376−378):

"~~This suggests adaptive levels of brain size variation, potentially underpinning behavioural diversity in division of labour, particularly when specific brain areas are considered.~~"

Reviewer Comment C3.4. Line 346: As with the comment above, there is no evidence provided in this paper that supports this statment. The paper cited–55–certainly is not appropriate here as it does not attempt at all to relate brain asymmetries with cognitive variability. The first part of this sentence should be removed.

Response. We agree and updated this statement in lines 462−465 as follows:

"This anatomical asymmetry could contribute ~~is consistent with~~ to the well-documented inter-individual cognitive variability in bumblebees [56~~5~~] and the fact that bees have better visual and olfactory learning abilities when stimuli are applied to right sensory organs [52~~1~~–55~~4~~]."

Reviewer Comment C3.5. Line 361: As with my comments above, there is no evidence provided in the paper that supports the statement that there is 'selection for variability supporting adaptive division of labour' and this part of the sentence should be removed.

Response. We agree and updated this statement in lines 499−501 as follows:

"[...]~~, suggesting selection for variability supporting adaptive division of labour~~. These anatomical differences could potentially generate behavioural variability and support adaptive collective behaviour, thereby calling for future studies in more species with contrasted social ecologies."

Reviewer Comment C3.6. Figure 5F: The y-axis does not show any numerical values apart from 0, please fix.

Response. Thanks for spotting this mistake that has been fixed.

Reviewer Comment C3.7. Figure 6: I do not find any information about the number of bees taken from each colony. Please provide this in the figure text.

Response. The number of bees per hive is given in S4 Table. We also added this information in the Materials and Methods section (lines 522, 523 and 526) as follows and now refer to this section in the legend of Fig. 6:

"Honey bees were collected from 9 hives located in two apiaries around Toulouse, France, in August 2020 (Population A (GPS coordinates: 43.55670, 1.47073): 100 bees from 6 hives: H1 (N=9), H2 (N=11), H3 (N=18), H4 (N=19), H5 (N=17), H6 (N=16); population B (43.26756, 2.28376): 20 bees from 3 hives: H7 (N=6), H8 (N=11), H9 (N=3)). Foragers returning to the colony were collected at the hive entrance, frozen and stored at -18°C. Bumblebees were purchased from a commercial supplier (Koppert, France) and 77 workers were collected from 4 hives~~colonies~~ and frozen at -18°C: H1 (N=18), H2 (N=20), H3 (N=21), H4 (N=18))."

Reviewer Comment C3.8. Figure 7: Separating the data from Rother et al. and Smith et al. is problematic as Rother et al. used data from Smith et al. so it is just a subset of their data and not a new data set. This should be removed. Also, it is not clear why this data is not presented as for the honey bees in Figure 5. I think that this should be done.

Response. Thank you for your feedback. We have removed Rother et al. from the comparison. Additionally, we have included two new figures in the Supporting Information (S9 and S10 Figs) illustrating the correlations of absolute and relative volumes of bumblebee neuropils with total brain volume, similar to Figs. 5 and S8 for honey bees, and we removed 7I and 7J from Fig 7 accordingly.

Reviewer Comment C3.9. Figure 8: it would be more informative if the correlations were made between the same individual rather than across the whole data set so a paired test should be used but this is not stated in the figure text.

Response. Thank you for your valuable comment. You are correct, and we confirm that a paired t-test was conducted. We have made the necessary updates to the figure legend as follows:

"[...] were obtained with Student's paired samples t-Test, and are displayed in bold when significant."

Have the authors made all data and (if applicable) computational code underlying the findings in their manuscript fully available?
The PLOS Data policy requires authors to make all data and code underlying the findings described in their manuscript fully available without restriction, with rare exception (please refer to the Data Availability Statement in the manuscript PDF file). The data and code should be provided as part of the manuscript or its supporting information, or deposited to a public repository. For example, in addition to summary statistics, the data points behind means, medians and variance measures should be available. If there are restrictions on publicly sharing data or code —e.g. participant privacy or use of data from a third party— those must be specified.

Reviewer #1: None

Reviewer #2: **No:** the raw volume data for individual 187 specimen are missing

Reviewer #3: Yes

PLOS authors have the option to publish the peer review history of their article (what does this mean?). If published, this will include your full peer review and any attached files.

If you choose "no", your identity will remain anonymous but your review may still be made public.

Do you want your identity to be public for this peer review? For information about this choice, including consent withdrawal, please see our Privacy Policy.

Reviewer #1: No

Reviewer #2: **Yes:** Jürgen Rybak

Reviewer #3: No

Figure Files:

While revising your submission, please upload your figure files to the Preflight Analysis and Conversion Engine (PACE) digital diagnostic tool, https://pacev2.apexcovantage.com. PACE helps ensure that figures meet PLOS requirements. To use PACE, you must first register as a user. Then, login and navigate to the UPLOAD tab, where you will find detailed instructions on how to use the tool. If you encounter any issues or have any questions when using PACE, please email us at figures@plos.org.

Data Requirements:

Please note that, as a condition of publication, PLOS' data policy requires that you make available all data used to draw the conclusions outlined in your manuscript. Data must be deposited in an appropriate repository, included within the body of the manuscript, or uploaded as supporting information. This includes all numerical values that were used to generate graphs, histograms etc.. For an example in PLOS Biology see here: http://www.plosbiology.org/article/info%3Adoi%2F10.1371%2Fjournal.pbio.1001908#s5.

Reproducibility:

To enhance the reproducibility of your results, we recommend that you deposit your laboratory protocols in protocols.io, where a protocol can be assigned its own identifier (DOI) such that it can be cited independently in the future. Additionally, PLOS ONE offers an option to publish peer-reviewed clinical study protocols. Read more information on sharing protocols at https://plos.org/protocols?utm_medium=editorial-email&utm_source=authorletters&utm_campaign=protocols

In compliance with data protection regulations, you may request that we remove your personal registration details at any time. (Remove my information/details). Please contact the publication office if you have any questions.
