# Supplementary material for: Natural variability in bee brain size and symmetry revealed by micro-CT imaging and deep learning
Source: PLoS Comput Biol. 2023 Oct 2;19(10):e1011529. doi: 10.1371/journal.pcbi.1011529 (PMC10569549; doi:10.1371/journal.pcbi.1011529)
Supplement: Attachment — Submitted filename: Response to Editors and Referees_REVIEW.pdf [file pcbi.1011529.s020.pdf]

Response to Editors and Referees
PCOMPBIOL-D-23-00086

Reviewer #1:

Thank the authors for following some of my suggestions although I still believe there are unresolved issues as indicated below in orange.

Reviewer Summary. The Manuscript "Natural variability in bee brain size and symmetry revealed by micro-CT imaging and deep learning" by Lösel et.al. is investigating the variations in total brain size of bees. To do so, the authors used semi-automated and fully automated methods to segment micro-CT scans of different bee specimens. Finally, the authors performed statistical analysis of the volumes of the bee brains and their inner parts.
It is a nice work which has various merits; however, the manuscript has some important issues that need to be revised before it can be considered for publication:

Reviewer Comment C1.1. In line 213, the authors claim that the dataset of their reference 29 is smaller though it is bigger. In Gronenberg -Couvillon publication the sample size of honeybees is 121.

Response. In reference 29, the total sample size of honey bees was indeed larger, but it was not homogeneous since it included samples taken from different subspecies i.e. 67 European and 54 Africanized honey bees, and the use of two bee strains may have led to biological differences in structural volumes, as discussed in lines 290−293. We have also clarified this point in lines 91−94 as follows:
"However, there is currently no clear connection between possible differences in brain neuro-architecture and behavioural variance due to the lack of data from comparative analyses (few studies [27] and sample sizes ranging from 7 [28] to 67 [29] individuals - [29] used 67 European honey bees and 54 Africanised honey bees)."

Reviewer Comment C1.2. The authors performed statistical analysis using 110 individuals of only one species of honeybees and 77 individuals of one species of bumblebees. Thus, it is a long stretch to generalize the results for all bee species, as there is a big variation between them. Therefore, I would suggest the authors to reconsider their title and be somewhat more precise/moderate.

Response. We agree that we cannot generalize our results on two model species (Apis mellifera and Bombus terrestris) to all bee species and made this clear in lines 497−501.

"Our results on two social bee species ~~honey bees and bumblebees~~ show high natural variability in brain area sizes that is stable across colonies, ~~suggesting selection for variability supporting adaptive division of labour~~. These anatomical differences could potentially generate behavioural

variability and support adaptive collective behaviour, thereby calling for future studies in more species with contrasted social ecologies."

However, we would like to keep using "bees" in the title instead of "Western honey bees and buff-tailed bumblebees" in order to keep it short.

Reviewer Comment C1.3. It is unclear whether the authors considered the age and the body size of the individuals as a factor during their statistical analysis. They mention that "... workers vary little in body size" but did they compare the body sizes of their dataset? Was the head size, eyes, or antenna size measured and compared with the associated neuropils? Also, was the age of all individuals taken as a parameter in the linear mixed model? Finally, I would expect logarithmic transformation of the comparative data, as ecological data are usually skewed to the right.

Response. Unfortunately, we did not have age and body size information and therefore could not add them as a factor in the statistical analyses. This was now clarified in lines 526–528:
"The analysed honey bees and bumblebees were of unknown age, and no body size information was available for these specimens."
We did not apply logarithmic transformation of our data because they were normally distributed as now mentioned in line 544 in the Methods section "Statistical analysis":
"Brain volumes were normally distributed."

Reviewer Comment C1.4. As we can see in table S3, the number of bees from each hive was quite different. We can see that even within this small size of hive sample, the variability is not trivial, especially for OTH. As the number of individuals in each hive sample is getting bigger so does the variability in brain volume. Thus, how can the authors generalize their results for all honeybees? How did the authors choose the fixed and random factors? Were the specific hives representatives of different features in honeybees? Was the population treated as numerical factor and if not, what was its threshold?

Response. Thank you for this comment. The level of variability within hives is indeed quite complex to interpret. As mentioned in the methods section "Sample preparation and CT scan" all our honey bees were from the same strain but sampled in two different apiaries. We have no information about the age of the bees nor their exact foraging experience or on potential external environmental stressors in the study area. Although our sample size is larger than in previous studies, we think that we do not have enough data and knowledge about our populations to fully understand the origin of this variability. We therefore prefer to remain descriptive and we have removed previous attempts to interpret this variability in evolutionary terms (see our response to Comment C3.3 by reviewer 3) or to generalize to all bee species (see our response to your Comment C1.2).

Reviewer Comment C1.5. The authors describe the statistical analysis of the variations within colonies but not the analysis between colonies.

Response. The statistical analysis between colonies is described in lines 545–548 which reads: "To analyse the inter-colonial variations of brain volume, we conducted linear mixed models (LMMs) using the lme4 package [686], with hive as fixed effect and population as random factor. LMMs were followed by F-tests to test the significance of fixed categorical variables using the anova function in the car package [697]."
See also lines 362–364:
"In our study, hHoney bees in the nine colonies exhibited overall similar average brain volumes (F-test: p=0.428; Fig 6A and Table 1), hence, inter-colony differences do not explain the substantial (32%) inter-individual variability."

Reviewer Comment C1.6. It is not very clear when the authors talk about sets of images, 3D images, 2D images, or scans; e.g., in line 134 they talk about different numbers of images, in line 141 about scans, in line 163 about 3D images. Even though they are all the same, it is better to keep one description for the readers' benefit.

Response. Thank you for your comment. To enhance clarity, we now use the terms "3D image", or "three-dimensional image" when it follows a numeric value, throughout the manuscript. In rare cases where we refer to a slice (i.e., a 2D image) extracted from a 3D image, we now explicitly indicate it.

Reviewer Comment C1.7. The authors mention in line 178 that their automatic segmentation is highly accurate, which is too subjective a statement. 12.5% of an error greater than 4% is higher than the 9.4% with very few errors in bumblebees. That, I believe, comes as a result of an overfitted network due to the lack of training data. I would suggest the authors used transfer learning algorithms to retain the weights from honeybees and refine them for the bumblebees. In that way their network will be more generalized.

Response. Thank you for this important comment. We have weakened the statement of the headline (lines 218–219) as follows:

"Accurate aAutomatic segmentation of bee brains with noticeable performance variations in bumblebees ~~is highly accurate~~"

While the segmentation accuracy is indeed lower in bumblebees compared to honeybees, it is important to note that this discrepancy arises from the lower image quality of the bumblebee data, rather than overfitting. To illustrate this, we have included S2 Fig, which showcases the segmentation results and errors of specific bumblebee images.
Regarding the size of training data, it is important to consider that we utilize 3D patches extracted from the 3D volumes, resulting in a substantial number of training patches. Each 3D image, scaled to 256x256x256 voxels, yields 343 patches of 64x64x64 voxels based on a stride size of 32 voxels. For the 13 volumetric training images of bumblebee brains, this amounts to a total of 4,459 individual training patches. These 3D patches are then utilized to train the neural network.

During inference, overlapping 3D patches of 64x64x64 voxels are employed to generate the 3D segmentation result. Both the standard accuracy and the Dice score stay close to their maximums (maximum standard accuracy after 154 epochs for bumblebees and after 197 epochs for honey bees, maximum Dice score after 129 epochs for bumblebees and after 123 epochs for honey bees) even up to 200 epochs (S4−7 Figs), indicating that overfitting is not a concern. Although, it is true that the loss function slightly starts to increase from around 100 epochs (S5 Fig). It is important to note that we always used Biomedisa's standard configuration to demonstrate what users can expect without optimizing parameters to their specific dataset. While transfer learning is particularly valuable when training data is limited, for the bumblebee dataset we conducted a comparison between fine-tuning the network trained on the honeybee dataset, following Amiri et al. 2020 (keeping the decoder part fixed and only training the encoder part), and training from scratch using different numbers of training images (S3 Fig). As expected, fine-tuning was better when the number of training images was small (3 to 7 training images) but not when more than 12 training images were utilized. It is worth noting that despite the similarity in brain shape, the bumblebee CT scans differ significantly from honeybee images. In particular in terms of contrast between brain areas, especially concerning CX (S2 Fig).

From Fig S4 we can see that after almost 100 epochs the training had no meaning, as the accuracy stayed the same and there is no significant difference in the test accuracy. Moreover, the figure is an indicator of overfitting, as the gap in accuracy of testing and training set is big. I would suggest the authors stopped the training after 100 epochs. Fig S5 also shows that after 100 epochs the network is overfitting, as the gap in loss is expanding.

Reviewer Comment C1.8. It is unclear why the authors chose 200 training epochs in such a small sample.

Response. The sample size, as mentioned earlier, is not small. Although the accuracy remains mostly unaffected, employing 200 epochs might prove inefficient. The decision to use 200 epochs was made to ensure thorough training of the network as now mentioned in lines 270−271. However, Biomedisa provides the option of early stopping to prevent unnecessarily prolonged training.

The training set is small considering the number of epochs. As stated in my previous comment, the presented training is not optimimal.

Reviewer Comment C1.9. Also, why the variation set is larger than the training set? How did the authors choose to split their data?

Response. The decision to have a larger validation set compared to the training set was made to align with the process used in analyzing our bee brains and what a typical Biomedisa user would do. We added the following paragraph to the "Increasing the number of 3D training images" section (lines 606−611):

"While the conventional practice often involves allocating approximately 80% of the data for training and 20% for testing, or using a split of 60% for training and 20% each for validation and testing, we opted for a training dataset size that aligns with the analysis procedure for our

specific bee brain dataset. Thus, we used up to 26 CT scans for training, selecting a similar amount for validation, considering the compute-intensive nature of validation during training, and assigned the remaining 3D images for testing."

Reviewer Comment C1.10. It is common to choose the values of hyperparameters based on the accuracy and performance of the network during training and validation. Can the authors provide such data?

Response. Biomedisa follows the idea of a simple and out-of-the-box tool. Therefore we chose the standard configuration of Biomedisa as described in the manuscript. They have proven to perform well on diverse image datasets. Although researchers can adapt the parameters to their own needs, we wanted to demonstrate the process based on the standard configuration without optimization making it simple to use. Nevertheless we checked the accuracy for varying learning rate, batch size, and network size and included this information in the new S2 Table. The accuracy for varying epochs can now be found in S4 & S6 Figs, which have been added to the Supporting Information and discussed in detail in the section "Accurate automatic segmentation of bee brains with noticeable performance variations in bumblebees". Most interestingly, choosing a smaller network size (up to 5 blocks with a maximum of 512 channels for the deepest layer) does not give statistically significant worse results but reduces computation time from 13 to 10 hours using 4 NVIDIA V100.

Even though I understand the Biomedisa contribution on this study, the focus of the specific paper is the use of deep learning methods for the segmentation of micro-CT images of bee brains. As such, Biomedisa should be a tool for pre-processing that can be skipped if someone chooses other manual segmentation methods for the creation of the masks. I think the authors should focus on the network and its optimization rather that choose the standard configuration of Biomedisa.

Reviewer Comment C1.11. How was the loss changing during training?
Response. The loss for the bumblebee dataset can be found in S5 Fig and the loss for the honey bee dataset in S7 Fig. The minima are after 34 epochs and 62 epochs, respectively.

Reviewer Comment C1.12. An ROC curve is essential to check the overfitting or underfitting of the network.
Response. The Receiver Operating Characteristic (ROC) curve is not typically used for measuring accuracy in image segmentation tasks. The ROC curve is commonly employed in binary classification problems where the goal is to distinguish between two classes, such as detecting whether an image contains a specific object or not. Image segmentation, on the other hand, involves dividing an image into meaningful regions or segments. Accuracy in image segmentation is typically evaluated using metrics that assess the quality of the segmentation masks, such as Intersection over Union (IoU), also known as the Jaccard index, or the Dice coefficient. These metrics measure the overlap between the predicted segmentation and the ground truth segmentation. The ROC curve is not directly applicable to segmentation because it requires a binary classification output. In image segmentation, the output is a pixel-wise mask

or a set of regions, rather than a binary classification label. To evaluate the performance of an image segmentation model, it is more appropriate to use metrics like IoU, Dice coefficient, or pixel-wise accuracy, which focus on the quality of the segmentation itself rather than classifying individual pixels as positive or negative.
The basic problem of applying ROC to image segmentation is that you take into account the background but you always have a huge amount of background labeled correctly resulting in AUC often of 1.0. In contrast, the Dice score does not consider the background but the overlap of a brain area with its ground truth making it much more reliable.

Image segmentation is an assignment of labels to pixels to create subgroups of the same important elements in the pixels. The output is a matrix of elements that specify the object class. Even though in the specific segmentation the authors chose multi-class segmentation, the ROC curve could be calculated using the different segmentation thresholds as in binary segmentations.

Reviewer Comment C1.13. In S1 Fig. it is unclear whether the network overpredicted the areas or mislabelled them. It would be much better if the authors provided the ground truth image too.
Response. We added the outline of the ground truth data to the right column of S1 Fig and added a similar figure for the bumblebee dataset (S2 Fig). While there is some overprediction, the errors occurring are most often mislabelling.

Reviewer Conclusion. Based on all these points, I think the manuscript needs to be significantly revised to warrant publication in PLOS Computational Biology.
